# Compulsory community treatment to reduce readmission to hospital and increase engagement with community care in people with mental illness: a systematic review and meta-analysis

**DOI:** 10.1016/S2215-0366(18)30382-1

**Published:** 2018-12

**Authors:** Phoebe Barnett, Hannah Matthews, Brynmor Lloyd-Evans, Euan Mackay, Stephen Pilling, Sonia Johnson

**Affiliations:** a Centre for Outcomes Research and Effectiveness, Department of Clinical Educational and Health Psychology, University College London, London, UK; b NIHR Policy Research Unit, University College London, London, UK; c Camden and Islington NHS Foundation Trust, London, UK

## Abstract

**Background:**

Compulsory community treatment (CCT) aims to reduce hospital readmissions among people with mental illness. However, research examining the usefulness of CCT is inconclusive. We aimed to assess the effectiveness of CCT in reducing readmission and length of stay in hospital and increasing community service use and treatment adherence.

**Methods:**

For this systematic review and meta-analysis, we searched three databases (PsycINFO, MEDLINE and Embase) for quantitative studies on CCT published in English between Jan 1, 1806, and Jan 4, 2018. We included both randomised and non-randomised designs that compared CCT with no CCT, and pre-post designs that compared patients before and after CCT. Studies were eligible if they had been peer-reviewed, if 50% or more of patients had severe mental illness, and if CCT was the intervention. Trials in which CCT was used in response to a criminal offence were excluded. We extracted data on study characteristics and length of follow-up, patient-level data on diagnosis, age, sex, race, and admission history, and outcomes of interest (readmission to hospital, inpatient bed-days, community service use, and treatment adherence) for meta-analysis, for which we extracted summary estimates. We used a random-effects model to compare disparate outcome measures and convert effect size statistics into standardised mean differences. This systematic review is registered with PROSPERO, number CRD42018086232.

**Findings:**

Of 1931 studies identified, 41 (2%) met inclusion criteria and had sufficient data for analysis. Before and after CCT comparisons showed significant large effects on readmission to hospital (standardised mean difference 0·80, 95% CI 0·53–1·08; *I*^2^=94·74), use of community services (0·83, 0·46–1·21; *I*^2^=87·26), and treatment adherence (2·12, 1·69–2·55; *I*^2^=0), and a medium effect on inpatient bed-days (0·66, 0·46–0·85; *I*^2^=94·12). Contemporaneous controlled comparison studies (randomised and non-randomised) showed no significant effect on readmission, inpatient bed-days, or treatment adherence, but a moderate effect on use of community services (0·38, 0·19–0·58; *I*^2^=96·92). A high degree of variability in study quality was found, with observational study ratings ranging from three to nine. Bias most frequently centred on poor comparability between CCT and control participants.

**Interpretation:**

We found no consistent evidence that CCT reduces readmission or length of inpatient stay, although it might have some benefit in enforcing use of outpatient treatment or increasing service provision, or both. Future research should focus on why some people do not engage with treatment offered and on enhancing quality of the community care available. Shortcomings of this study include high levels of variability between studies and variation in study quality.

**Funding:**

National Institute for Health Research.

## Introduction

Compulsory community treatment (CCT) makes adherence to treatment a legal requirement for people with mental illness who live in the community.^1^ The introduction of CCT in England and Wales, the USA, Australia, the Netherlands, Sweden, Spain, and parts of Canada^1^, ^2^ has triggered substantial debate about whether such measures reduce so-called revolving door use of inpatient services enough to justify the associated restriction of patient liberty.^3^ In England and Wales, CCT makes patients who are discharged from compulsory inpatient care subject to hospital recall if they do not comply with conditions that can include maintaining contact with services.^3^, ^4^ CCT can also require adherence to conditions including taking medication, abstaining from illicit drugs, and attending outpatient appointments.^5^ CCT can also impose requirements on service providers to make care available.^1^ CCT schemes differ among countries in terms of length, extent, and prerequisites of compulsion.^6^

Arguments for CCT concentrate on its less restrictive nature compared with admission to hospital,^1^, ^3^, ^7^ and its potential to prevent relapses and readmissions.^8^ However, evidence supporting these benefits is inconclusive and CCT could risk replacement of engagement and building therapeutic alliance with unjustified control and threat, while ignoring underlying reasons for non-adherence^7^, ^9^—eg, the need for better community services or increased engagement of service users in decision making.^3^, ^10^ Furthermore, restriction of liberty of adults who might have capacity for decision making and who have broken no laws has been questioned by both service users and health-care professionals.^8^, ^11^ However, some studies have reported preference among patients for CCT if the alternative is admission to hospital,^12^ and carers also support use of CCT.^13^, ^14^ The benefits of CCT might also result from community services being obliged to provide care instead of any direct effect on patient behaviour.^15^

**Research in context**

**Evidence before this study**

Compulsory community treatment (CCT) allows a legal requirement to be put in place for patients to maintain contact with mental health services or receive treatment in the community, or both. Evidence from the few trials done in this field suggest that CCT has little to no effect on outcomes, including admissions, but critiques of these trials suggest they might have a high risk of selection bias. A larger body of evidence exists from observational studies, and inclusion of these studies in decision making and analysis could have the advantages endowed by larger and more representative cohorts. We searched PsycINFO for systematic reviews and meta-analyses published in English between Jan 1, 1806, and Dec 31, 2017, using the search terms “community treatment order” or “CTO” or “Outpatient commitment” or “Civil commitment” and “meta-analys*” or “metasynthes*” or “meta-synthes*”. The search returned ten articles, three of which were relevant to our review (Maughan and colleagues [2014], Rugkåsa [2016], and Kisely [2107]); however, a quantitative synthesis of all quantitative designs on the effectiveness of CCT had not been done.

**Added value of this study**

To our knowledge, this is the first systematic review and meta-analysis of all quantitative randomised and non-randomised studies on CCT to date. We include both contemporaneous comparisons between patients released on CCT and those without CCT, and comparisons of patients before and after CCT. Before and after comparisons showed improvements across all outcomes, but contemporaneous comparisons (both randomised and non-randomised) showed little to no effect on readmission to and length of stay in hospital, or treatment adherence. In contemporaneous comparisons, some evidence supported increased use of community services with CCT, suggesting the treatment might be effective in increasing service provision or treatment attendance, or both. Treatment adherence was not consistently reported. Therefore, our results are mixed, but more methodologically robust designs indicate that CCT does not, as intended, reduce readmissions to or length of stay in hospital.

**Implications of all the available evidence**

Our findings do not clearly support CCT in the prevention of repeat admissions to hospital. Alternative methods of reducing repeat admission of compulsorily detained patients should be investigated—eg, by investing in more and better admission alternatives or community services on discharge than are currently available.

The ethics and effectiveness of CCT continue to be debated, with previous systematic reviews finding that CCT is of uncertain benefit.^1^, ^4^, ^8^, ^16^ A Cochrane review and meta-analysis of three randomised trials^16^ showed little to no effect of CCT compared with standard voluntary care. However, some researchers argue that participants entering these trials are likely to be unrepresentative of the population for whom the treatment is intended.^5^ For example, in the UK OCTET trial,^17^ psychiatrists giving treatment could exclude patients whom they felt would clearly benefit from CCT and found both treatment groups to be largely adherent to treatment at follow-up, suggesting people in randomised trials are not representative of the patients who do not engage with treatment for whom CCT is intended. The Cochrane review and meta-analysis^16^ found that the included randomised trials had major limitations resulting from difficulties in doing trials in a population who do not wish to cooperate and could pose a high risk; small samples, few trials, and selection bias were identified as key limitations in the validity of interpretations drawn from such trials. Thus, considering epidemiological studies of unselected clinical samples alongside these trials could enable improved analyses and understanding.

We aimed to update and quantify current knowledge of the effectiveness of CCT in reducing readmission to and length of stay in hospital, and increasing use of community services and treatment adherence for patients with severe mental illness. We include a substantial body of evidence from observational studies. Although these studies are more susceptible than randomised controlled trials to bias from differences between groups, they offer the advantage of improved representativeness of individuals undergoing CCT both nationally and internationally.

## Methods

### Search strategy and selection criteria

In this systematic review and meta-analysis, we developed a search strategy based on strategies used by Kisely and colleagues^16^ and Rugkåsa^8^ using keyword and subject searches containing generalised mental health and CCT-specific terms. This review adhered to the Preferred Reporting Items for Systematic reviews and Meta-Analysis (PRISMA) guidelines.^18^ We developed a review protocol and adhered to it throughout the review process. We searched three electronic databases (PsychINFO, for articles published between Jan 1, 1806, and the fourth week of December, 2017; Embase, between Jan 1, 1974, and the first week of January, 2018; and MEDLINE, between Jan 1, 1946, and the fourth week of January, 2018) for publications in English, using the search terms “community treatment order” or “CTO” or “outpatient commitment” or “‘compulsory’ or ‘mandatory’ outpatient commitment” or “civil commitment” AND “SMI” or “psychiatric” or “manic” or “schizophrenia” or “bipolar”. We then applied a backwards reference search to the studies identified by manually searching reference lists of eligible studies. We also searched for articles that cited eligible studies using Scopus, and assessed those for eligibility. We searched review articles identified through the search to identify additional studies; however, no further studies were found (full search strategy is in the appendix).

Inclusion criteria were peer-reviewed studies with samples in which the majority (>50%) of patients had severe mental illness, and peer-reviewed studies with CCT interventions, defined as legal compulsion on patients to remain in contact with mental health services or accept treatment in the community, or both. Interventions in which compulsion was in response to a criminal offence were excluded. We defined comparative treatments as those that did not consist of compulsory treatment in the community; the comparison did not have to be an alternative treatment, it could also have been no treatment at all (ie, release without compulsory treatment). Our primary outcome measure of interest was readmission to hospital. Secondary outcome measures of interest were length of hospital stay (ie, inpatient bed-days), use of community services, and treatment adherence. We extracted data on readmission to hospital regardless of whether the readmission was specified as compulsory or not, under the assumption that it was compulsory in each study, although some legal health-care frameworks (eg, in England) allow exceptions. Eligible study designs were quantitative randomised controlled trials, contemporaneous controlled comparison studies comparing a group who were subject to CCT with a group not subject to CCT, and pre-post studies comparing service use by patients before and after the imposition of CCT. A summary of the study protocol is available online.

### Data extraction and quality assessment

Two reviewers (PB, EM) screened the abstracts of all studies identified through the initial search and excluded those that did not meet the inclusion criteria. Full-text articles of eligible studies were then obtained and reviewed in duplicate, with conflicts resolved by discussion and where necessary consultation with a third reviewer (HM). PB and EM extracted data on study design, sample size, country, diagnosis, age, sex, race, admission history, length of follow-up, and outcome measures of interest with associated statistical data (ie, odds ratio or events data) using an electronic Microsoft Excel-based form. For outcomes of interest, we extracted summary estimates rather than individual-level data.

To assess the quality of observational studies, two raters (PB, EM) used the Newcastle-Ottawa scale. This scale offers quality assessment scales for cohort and case-control studies. The raters independently applied the scale to each study and discrepancies were resolved by discussion or consultation with a third reviewer (HM) if an agreement could not be reached. This nine point scale can be divided into three categories with a maximum number of items for each category: selection of study groups (four items), comparability of the groups (two items), and ascertainment of exposure or outcome of interest (three items). Studies were awarded one point for each item within selection and exposure categories, and up to two points for items in the comparability category. Each study's score was calculated by summing the total of these scores. Studies with a score of six or more were considered high quality, and studies with a score of less than six were considered low quality.^19^ To assess the methodological quality of randomised controlled trials, we used the Cochrane Risk of Bias Tool.^20^ We rated selection, performance, detection, attrition, and reporting bias as unclear, low, or high risk for each study.

### Data analysis

We did a meta-analysis to compare disparate outcome measures between studies. To allow this comparison, we converted effect size statistics to standardised mean differences with 95% CIs using a random-effects model. This model assumes that the analysed studies are a random sample of effect sizes, enabling the generalisability of results,^21^ and we considered it appropriate for examining studies from a range of countries with differing CCT specifications. We included only unadjusted data in our analyses. We excluded publications that only provided data that we had already extracted. Data from studies with contemporaneous control groups and with before and after (pre-post) CCT designs were analysed separately. We considered results to be significant if their p value was less than 0·05. We did not apply corrections for multiple testing, but all tests were reported. We calculated heterogeneity between studies using *I*^2^. A value of 0% indicates no observed heterogeneity, 25% low heterogeneity, 50% moderate heterogeneity, and 75% high heterogeneity.^22^ To minimise heterogeneity, we analysed data recorded at 12 months of follow-up. For studies that did not report data at 12 months, we selected the timepoint at which data were collected that was closest after 12 months, or before 12 months for shorter follow-ups. We used conventional values of effect size,^23^ with values around 0·2 indicating a small effect, 0·5 a moderate effect, and 0·8 a large effect. We separately analysed studies comparing the same sample before and after CCT and studies comparing patients on CCT to controls. For studies that reported both comparisons, we extracted and analysed data for both. We assessed the degree of publication bias (ie, the preferential publication of studies with positive effects) on the mean effects of each outcome by visual examination of a funnel plot. We did two sensitivity analyses to test a priori assumptions that inclusion of only primary outcomes or adjusted data might influence our findings. The first analysis included only primary outcomes as identified by the authors of the study and the second included adjusted data when unadjusted data were unavailable. We did four meta-regressions on publication year, follow-up duration, study quality, and country on the main outcome of readmission to hospital. We also planned to investigate outcomes of CCT for children, women, and ethnic groups.

We did our analyses using Comprehensive Meta-Analysis software (CMA Version V3). This review was prospectively registered with PROSPERO, number CRD42018086232.

### Role of the funding source

The funder of the study had no role in study design, data collection, data analysis, data interpretation, or writing of the report. The corresponding author had full access to all the data in the study and had final responsibility for the decision to submit for publication.

## Results

**Figure 1:**
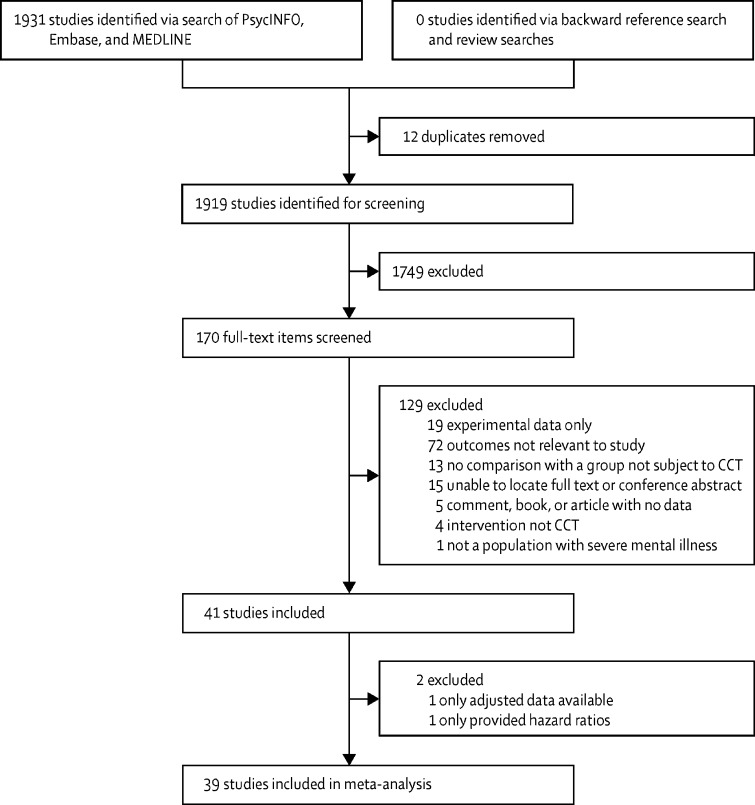
Study selection CCT=compulsory community treatment.

**Figure 2:**
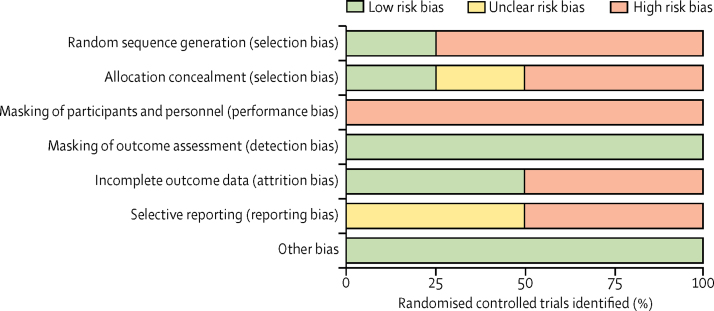
Risk of bias Reviewers' judgement about each risk of bias item as a proportion of all included randomised controlled trials.

Of 1931 studies found, 170 (9%) were identified as potentially relevant full-text articles and were reviewed for eligibility (figure 1). 41 (24%) of 170 studies provided sufficient data comparing CCT with controls or comparing data recorded after the intervention to data recorded before the intervention for our selected outcomes, and were included in our systematic review (n=189 749); 39 studies (n=57 746) were included in our meta-analysis (two studies were excluded for not having data in the correct format for analysis). Study characteristics for all 41 studies are summarised in table 1, and risk of bias for randomised controlled trials is shown in figure 2. Of 41 studies, unadjusted data were available on readmission to hospital in 30 (73%), inpatient bed-days in 26 (63%), community services in 14 (34%), and treatment adherence in five (12%). One study^41^ did not have unadjusted data on outcomes of interest, and so it was not included in the main meta-analysis. One study^28^ reported only hazard ratios, which could not be combined with other studies in a meta-analysis, so was excluded. Studies with a pre-post design most frequently followed up patients before and after they were put on CCT, although some provided population statistics before and after the introduction of CCT legislation. In all studies comparing CCT with a control group, all comparisons assumed a control of patients who had been voluntarily discharged, except for two studies: one study used extended leave as a comparator^24^ and one study used patients who remained under section in hospital as a comparator.^30^ We saw a high degree of variability in study quality, with ratings for observational studies ranging from three to nine. Bias as measured by the study quality rating most frequently concentrated on poor comparability between CCT and control participants.

**Table 1:** Study characteristics

	**Sample size**	**Outcomes reported**	**Country**	**Mean age, years (range)**	**Sex (%)**		Length of follow-up*	**Newcastle-Ottawa score**
					Female	Male		
**Randomised controlled trial**								
Burns et al (2013)^24^	336	Readmission to hospital; inpatient bed-days; use of community services	England, UK	39·6 (18–65)	34%	66%	12 months	NA
Wagner et al (2003)^25^	264	Use of community services	USA	NR	NR	NR	12 months	NA
Steadman et al (2001)^26^	142	Readmission to hospital	USA	41 (NR)	31%	69%	11 months	NA
Swartz et al (1999)^27^	264	Readmission to hospital; inpatient bed-days	USA	39·6 (NR)	50%	50%	12 months	NA
**Case-control study**								
Van Dorn et al (2010)^15^	3576	Readmission to hospital; treatment adherence	USA	41·8 (NR)	32%	68%	88 months	6
**Cohort study**								
Burgess et al (2006)†^28^	128 427	Readmission to hospital	Australia	40·1 (NR)	47%	53%	96 months	5
Bursten (1986)^29^	156	Readmission to hospital	USA	35·9 (NR)	36%	64%	14 months	4
Castells-Aulet et al (2015)^30^	150	Readmission to hospital; inpatient bed-days	Spain	41·6 (NR)	33%	66%	24 months	6
Geller et al (1998)^31^	19	Readmission to hospital; inpatient bed-days	USA	38·5 (NR)	37%	63%	6 months	5
Hernández-Viadel et al (2010)^32^	76	Readmission to hospital	Spain	41·5 (NR)	32%	68%	6 months	5
Hiday and Scheid-Cook (1987)^33^	7002	Readmission to hospital; use of community services; treatment adherence	USA	NR	43%	57%	6 months	3
Hiday and Scheid-Cook (1989)^34^	740	Readmission to hospital; use of community services	USA	NR	41%	59%	6 months	5
Kisely et al (2005)^35^	392	Readmission to hospital; inpatient bed-days	Australia	37·2 (NR)	35%	65%	12 months	6
Kisely et al (2004)^36^	754	Readmission to hospital	Australia	37·4 (NR)	36%	64%	12 months	8
Pollack et al (2005)^37^	290	Readmission to hospital; use of community services	USA	42 (NR)	47%	53%	36 months	5
Segal and Burgess (2006a)^38^	4146	Readmission to hospital; inpatient bed-days; use of community services	Australia	30·3 (NR)	35%	65%	6 months	6
Segal and Burgess (2006b)^39^	24 973	Readmission to hospital; inpatient bed-days; use of community services	Australia	44·2 (NR)	44%	56%	120 months	7
Segal et al (2009)^40^	246	Use of community services	Australia	33·9 (NR)	35%	65%	12 months	8
Swartz et al (2010)‡^41^	3576	Readmission to hospital; use of community services; treatment adherence	USA	41·8 (NR)	32%	68%	96 months	5
**Pre-post study design**								
Awara et al (2013)^42^	34	Readmission to hospital; inpatient bed-days	England, UK	45 (NR)	32%	68%	6 months	6
Castells-Aulet et al (2013)^43^	91	Readmission to hospital; inpatient bed-days	Spain	41 (22–71)	33%	63%	24 months	8
Christy et al (2009)^44^	50	Readmission to hospital	USA	41 (19–NR)	52%	48%	24 months	8
Dye et al (2012)^45^	21	Readmission to hospital; treatment adherence	England, UK	50 (NR)	45%	55%	24 months	8
Erickson (2005)^46^	100	Readmission to hospital	USA	36·6 (18–65)	43%	57%	18 months	8
Fernandez and Nygard (1990)^47^	4179	Readmission to hospital; inpatient bed-days	USA	36·8 (NR)	41%	59%	36 months	8
Kallapiran et al (2010)^48^	28	Readmission to hospital; inpatient bed-days	USA	NR	19%	81%	12 months	9
Kijellin and Pelto-Piri (2014)^49^	1038	Readmission to hospital; inpatient bed-days	Sweden	NR	50%	50%	24 months	8
Lera-Calatayud et al (2014)^50^	140	Readmission to hospital; inpatient bed-days	Spain	41 (21–75)	34%	66%	12 months	8
Muirhead et al (2006)^51^	94	Readmission to hospital; inpatient bed-days; use of community services	USA	39·4 (18–66)	30%	70%	12 months	9
Munetz et al (1996)^52^	20	Readmission to hospital; inpatient bed-days; use of community services	USA	41·4	45%	55%	12 months	8
O'Brien and Farrell (2005)^53^	NR	Readmission to hospital; inpatient bed-days; use of community services	Canada	45 (20–70)	40%	60%	12 months	8
O'Brien et al (2009)^54^	84	Use of community services	Canada	42 (16–76)	40%	60%	12 months	7
O'Keefe et al (1997)^55^	26	Inpatient bed-days; treatment adherence	USA	43 (NR)	46%	54%	24 months	6
Ozgul and Brunero (1997)^56^	46	Readmission to hospital; inpatient bed-days	Australia	36 (NR)	33%	67%	36 months	7
Rawala and Gupta (2014)^57^	37	Treatment adherence	England, UK	40·9 (25–65)	8%	92%	6 months	7
Rohland et al (2000)^58^	81	Readmission to hospital; inpatient bed-days	USA	36·9 (18–76)	38%	62%	60 months	8
Taylor et al (2016)^59^	1558	Inpatient bed-days	Scotland, UK	NR	37%	63%	12 months	7
**Cohort study with pre-post design**								
Hunt et al (2007)^60^	316	Readmission to hospital; inpatient bed-days; use of community services	Canada	NR	55%	45%	12 months	6
Kisely et al (2013)^61^	5916	Inpatient bed-days; use of community services	Australia	36·8 (NR)	36%	64%	12 months	5
Vaughan et al (2000)^62^	246	Readmission to hospital; inpatient bed-days	Australia	36·1 (NR)	32%	68%	24 months	7
Zanni and Stavis (2007)^63^	115	Readmission to hospital; inpatient bed-days	USA	NR	NR	NR	24 months	8

NA=not applicable. NR=not recorded.

* Maximum follow-up reported in study; however, 12 month data was extracted for analysis when available, or data closest to 12 months when not.

† Only hazard ratios were provided, which could not be combined with other outcomes, therefore this study was excluded from the main meta-analysis.

‡ Only adjusted data were provided, therefore this study was not included in the main meta-analysis.

Effect sizes with 95% CIs from pre-post studies are shown in table 2. 17 studies had before and after CCT comparisons and four studies had both before and after and control group comparisons (relevant data from these studies were included separately in each analysis), including 9455 participants from six countries (England, Scotland, Australia, the USA, Canada, and Spain). We saw a medium effect for reduction in inpatient bed-days (standardised mean difference 0·66, 95% CI 0·46–0·85), and a large effect for reduction in readmission to hospital (0·80, 0·53–1·08), increase in use of community services (0·83, 0·46–1·21), and increase in treatment adherence (2·12, 1·69–2·55).

**Table 2:** Comparison of outcomes from pre-post studies of compulsory community treatment

	**Number of studies**	**Effect size (95%CI)**	**p value**	**Heterogeneity (I^2^)**
Readmission to hospital	14	0·80* (0·53–1·08)	<0·0001	94·74
Readmission to hospital (rates)†	2	0·75‡ (0·47–1·20)	0·2332	99·71
Inpatient bed-days	14	0·66* (0·46–0·85)	<0·0001	94·12
Use of community services	6	0·83* (0·46–1·21)	<0·0001	87·26
Treatment adherence	3	2·12* (1·69–2·55)	<0·0001	0

* Standardised mean difference, random-effects model.

† Two studies reported readmission data as rate ratios, which cannot be combined with other data formats, so are reported as a separate analysis.

‡ Rate ratio, random-effects model.

**Figure 3:**
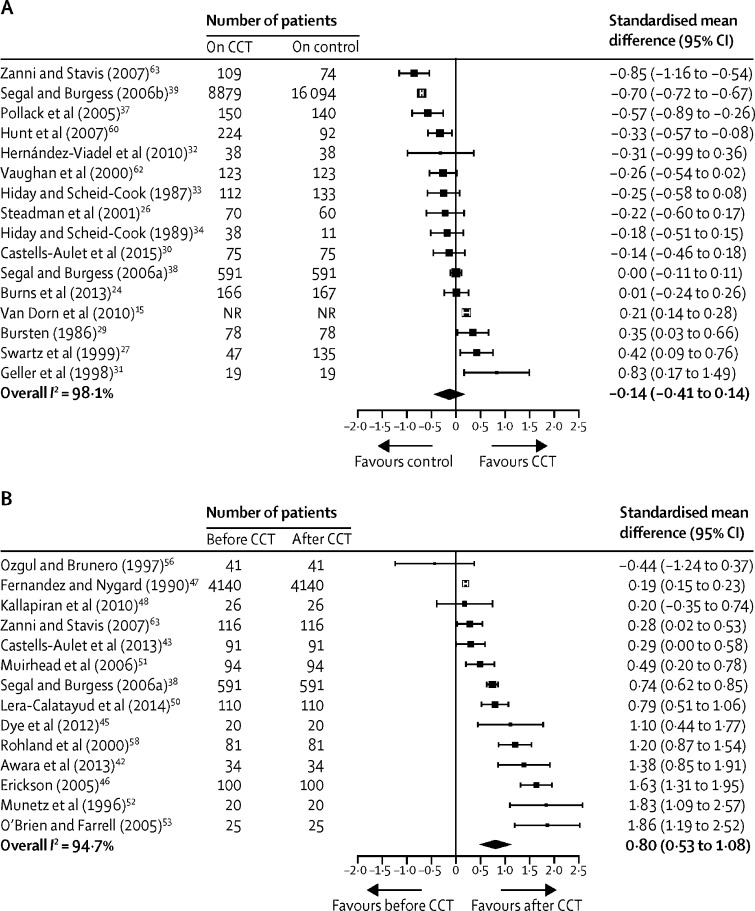
Effect on readmission to hospital for CCT versus control (A) and for before versus after CCT (B) Boxes are point estimates, with error bars for 95% CIs. CCT=compulsory community treatment. NR=not reported.

Effect sizes for contemporaneous comparisons of patients on CCT with controls are shown in table 3. 20 studies had this design, of which 16 were non-randomised and four were randomised studies, and four studies had both before and after and control group comparisons. These studies included 181 150 participants from five countries (England, Australia, the USA, Canada, and Spain). No effect was seen in reducing readmission to hospital (standardised mean difference −0·14, 95% CI −0·41 to 0·14) or inpatient bed-days (0·13, −0·08 to 0·34), a moderate effect was seen for increase in use of community services (0·38, 0·19 to 0·58), and a large but non-significant effect was seen for increase in treatment adherence (0·91, −0·70 to 2·51). Figure 3 shows forest plots for readmission to hospital for both before and after and control comparisons**;** plots for other outcomes of interest and for analyses of studies in which rates (ie, risk ratios for the control comparison, and rate ratios for the pre-post comparison) were reported for readmission are presented in the appendix.

**Table 3:** Effect on outcome of compulsory community treatment compared with controls

	**Number of studies**	**Effect size (95% CI)**	**p value**	**Heterogeneity (I^2^)**
Readmission to hospital	17	−0·14* (−0·41 to 0·14)	0·3358	98·06
Readmission to hospital (rates)†	2	1·98‡ (0·78 to 5·01)	0·1509	94·11
Inpatient bed-days	11	0·13* (−0·08 to 0·34)	0·2335	97·18
Use of community services	9	0·38* (0·19 to 0·58)	0·0001	96·92
Treatment adherence	2	0·91* (−0·70 to 2·51)	0·2677	87·93

Control was no compulsory community treatment.

* Standardised mean difference, random-effects model.

† Two studies reported readmission data as risk ratios, which cannot be combined with other data formats and so are reported as a separate analysis.

‡ Risk ratio, random-effects model.

Reduction in readmission to hospital remained non-significant when separately analysing randomised controlled trials (three studies, standardised mean difference −0·08, 95% CI −0·26 to 0·41), and non-randomised studies (14 studies, −0·18, −0·49 to 0·12). Reduction in inpatient bed-days also remained non-significant when separately analysing randomised controlled trials (two studies, 0·25, −0·11 to 0·61) and non-randomised studies (nine studies, 0·10, −0·14 to 0·33). Increase in use of community services remained significant when non-randomised studies were analysed separately (seven studies, 0·46, 0·24 to 0·67) but became non-significant when only randomised controlled trials were analysed (two studies, 0·13, −0·03 to 0·29; appendix).

Both sensitivity analyses resulted in only a marginal change in effect sizes for each outcome, except for treatment adherence for which the non-significant effect was reduced (Table 4, Table 5).

**Table 4:** Sensitivity analysis of primary outcome measures, as identified by study authors

	**Number of studies**	**Comparison**	Original effect size*(95% CI)	New effect size*(95% CI)
Readmission to hospital	12	CCT *vs* no CCT	−0·14 (−0·41 to 0·14)	−0·12 (−0·41 to 0·17)
Readmission to hospital	11	Before *vs* after CCT	0·80 (0·53 to 1·08)	0·59 (0·34 to 0·84)
Use of community services	3	Before *vs* after CCT	0·87 (0·42 to 1·33)	1·02 (0·54 to 1·51)

Analysis was only done for outcome measures that were identified as the primary outcome measure in three or more studies. CCT=compulsory community treatment.

* Standardised mean difference.

**Table 5:** Sensitivity analysis of adjusted data

	**Number of studies**	**Comparison**	Original effect size*(95% CI)	New effect size*(95% CI)
Readmission to hospital	17	CCT *vs* no CCT	−0·14 (−0·41 to 0·14)	−0·11 (−0·40 to 0·18)
Use of community services	9	CCT *vs* no CCT	0·38 (0·19 to 0·58)	0·43 (0·29 to 0·58)
Treatment adherence†	3	CCT *vs* no CCT	0·91 (−0·70 to 2·51)	0·30 (0·13 to 0·48)

Data in parentheses are 95% CIs. CCT=compulsory community treatment.

* Standardised mean difference.

† Effect size for treatment adherence decreases substantially, although this result should be interpreted with caution because this analysis only includes three studies.

In our meta-regressions for readmission to hospital, year of publication was a significant moderator for both before and after CCT (11 studies, *R*^2^=0·49; p=0·0022) and control comparisons (13 studies, *R*^2^=0·71; p=0·0002; appendix). Although different publication years predicted different effects of CCT on readmission to hospital, no consistent trend over time was observed. Duration of follow-up was not a significant moderator of before and after CCT comparisons (11 studies, *R*^2^=0·00; p=0·73), but it was significant for comparison of CCT with a control (13 studies, *R*^2^=0·20; p=0·0228). Country of study was not a significant moderator for before and after CCT (11 studies, *R*^2^=0·00; p=0·3574) or control comparisons (13 studies, *R*^2^=0·00; p=0·9479). Study quality was not a significant moderator for before and after comparisons (11 studies, *R*^2^=0·00; p=0·5628) or control comparisons (13 studies, *R*^2^=0·16; p=0·1013). Scatter plots for meta-regressions are in the appendix. There was insufficient data to analyse outcomes in only children, only women, or according to ethnicity. Visual examination of the funnel plots showed little publication bias in our estimate of CCT effectiveness (appendix). A high level of heterogeneity was observed for readmission to hospital, inpatient bed-days, and use of community services, and a low level of heterogeneity for treatment adherence (Table 2, Table 3).

## Discussion

In this meta-analysis we found that CCT does not have a clear positive effect on readmission and use of inpatient beds. Evidence suggested a potentially positive effect on treatment adherence, although this result should be interpreted with caution because of the small number of studies included in the analysis. Although CCT might result in increased community service availability, in the absence of clear, consistent evidence on clinical benefits, and the removal of patient liberty involved, this effect is probably insufficient to justify use of CCT. Our review suggests a need to critically examine the future justification for CCT.

In this systematic review and meta-analysis we provide an updated synthesis of the available evidence for all empirical research on, and quantify the effectiveness of, CCT. Existing evidence has suggested little to no effect, calling into question the reasoning for implementation of CCT in policy.^1^, ^4^, ^8^ Our meta-analysis was broader than previous analyses because, in addition to randomised controlled trials, we included other study designs, such as non-randomised designs that can have less selection bias and provide larger samples than randomised trials. We analysed studies with contemporaneous control groups, and studies with a before and after (pre-post) CCT design separately. The emerging results varied substantially, with high levels of heterogeneity observed across outcome measures despite our attempts to separate designs. This heterogeneity was probably a result of varying policies, ethnicities, sexes, ages, and treatments in the international studies included, which further complicated the task of disentangling the results. Before and after studies suggested that patients are substantially less likely to be readmitted to hospital after CCT than before CCT. However, the particular problems associated with designs of this type have been frequently noted in the methodological literature,^64^ and include regression to the mean and maturation effects. For example, if CCT is used at the end of a long stay in hospital, subsequent improvement could simply reflect regression to the clinical mean due to the apparent cyclical nature of illness severity.^16^ Thus, overall we believe the conservative view that CCT does not clearly show benefits is supported by the evidence, which is a view supported by randomised controlled trials and observational studies in which contemporaneous comparisons have been made, although the potential effects of confounding in natural experiments are an important limitation that should be considered.

Our meta-regression analyses identified two significant covariates: duration of follow-up and publication date. These factors account for a proportion of variance in readmission to hospital; however, no evidence exists to support a clear pattern for these covariates—eg, our evidence does not suggest that CCT produces better outcomes in studies published more recently or in studies with a longer follow-up period. Study quality varied substantially; however, our meta-regressions did not find an association between study quality and outcome. Overall, our findings indicate patients on CCT used more community services after discharge from hospital than those not on CCT and than they did before CCT; however, this finding could indicate that people on CCT were offered more services than others. This finding raises the question of whether compulsion is necessary if indeed it does not reduce readmission to hospital compared with no CCT.^3^, ^10^ Only a few studies^15^, ^33^, ^41^, ^45^, ^55^ have reported adherence to treatment; hence, this outcome measure requires increased attention in future empirical research because it is central to arguments for compulsion in community treatment.^1^

This study has several limitations. Most studies included in our analysis were observational, with a non-randomised comparison of CCT with either a non-compulsory group or the time before admission to hospital, both of which are problematic for group comparability. The heterogeneity observed could be a result of this issue because patients who are severely ill are more often released with compulsory orders than without such orders.^39^ Randomised controlled trials have attempted to overcome this problem, but only four have been published to date, and difficulties regarding ethical considerations and problems with overly selected samples that might not be representative of the target population remain a key hurdle.^17^ Therefore, we believe interpretation of findings from an overview of both randomised and non-randomised evidence to be preferable to either in isolation. Another limitation was that insufficient data were available to do prespecified subgroup analyses to investigate outcomes of CCT for children, women, and ethnic groups. Analyses in these populations have been specifically recommended by the National Institute for Health Research Lived Experience Working Group as a focus for future work (panel). Also, two studies could not be included in the meta-analysis because they did not report unadjusted or compatible data,^28^, ^41^ and the required data could not be obtained from the authors. Although we recognise the argument for the use of adjusted data to control for other potential moderators of mental health outcomes, we used unadjusted data in our analyses because only a few studies that were identified as eligible reported adjusted data, and those studies had large differences in the number and types of variables controlled. However, our sensitivity analysis that included adjusted data did not give substantially different results from the main analyses. Despite this fact, our analyses should be interpreted with the understanding that several other possible variables—eg, diagnosis, ethnicity, comorbidities, country, and criminal convictions^6^, ^36^, ^65^—could also have a key role in the effect of CCT. Additionally, other important outcomes, particularly associated with patient experience and risk (eg, number of deaths or negative experience) could be relevant to policy making regarding CCT, but were insufficiently reported in the literature for inclusion in our analyses.

Panel **Message from the National Institute for Health Research Lived Experience Working Group**

As service users and carers, we are not inspired by measures of readmissions and inpatient bed-days; we want to know what difference interventions make to the quality of people's lives and wellbeing. Although data on such outcomes are disappointingly missing from the literature, the evidence that researchers have analysed in this systematic review still paves the way for a prompt review of the use of compulsory community treatments (CCTs). We hope that future work will address the glaring omissions in existing data that have been highlighted by this study. Ethnicity is a fundamental aspect that influences the disproportionate use of CCTs in specific groups. As increased emphasis is placed on a biopsychosocial model, data on accommodation, employment, and community networks need to be included, alongside the role of carers and the effect on family. We are also interested in who is subject to CCT. We want to see a fuller picture of the experiences of specific groups, including black African Caribbean men, women labelled with personality disorders, and young people with learning disabilities. For the moment, this study, like all previous studies, suggests that CCTs have little effect on inpatient services, but have some effect on the use of community services. But would coercion be needed if people could easily access appropriate community services? And also, how many studies coming to the same conclusion does it take before action is taken?

Our findings in this review have important implications for practice and policy. We suggest that evidence of improvement in patient outcomes after CCT compared with before inpatient admission is insufficient without additional consistent evidence for their effectiveness in reducing readmission to hospital through more methodologically robust designs. Additionally, the potential increased cost and coercive nature of CCT is difficult to justify without more consistent evidence. Alternative investments in providing more accessible, high quality community support than currently available could result in increased benefits—eg, advance directives and joint crisis plans have shown some benefits in reducing compulsory admissions.^66^ Other alternative approaches could involve enhancing the quality of inpatient care—such as improving understanding of why some people do not engage with the treatment that they are offered and what might make treatment more acceptable, and interventions designed with service users focused on engaging and preventing relapse among those with a history of compulsory admission. Some clinical groups might also benefit more from CCTs than others—eg, those with more severe illness who are typically excluded from randomised controlled trials and who might have contributed to our significant pre-post findings. The increasing availability of large routine datasets could allow this avenue to be explored in future.

For the **protocol** see https://www.crd.york.ac.uk/prospero/display_record.php?RecordID=86232
