# Supplementary material for: Compulsory community treatment to reduce readmission to hospital and increase engagement with community care in people with mental illness: a systematic review and meta-analysis
Source: Lancet Psychiatry. 2018 Dec;5(12):1013–22. doi: 10.1016/S2215-0366(18)30382-1 (PMC6251967; doi:10.1016/S2215-0366(18)30382-1)
Supplement: Supplementary appendix [file mmc1.pdf]

THE LANCET
Psychiatry

Supplementary appendix

This appendix formed part of the original submission and has been peer reviewed. We post it as supplied by the authors.

Supplement to: Barnett P, Matthews H, Lloyd-Evans B, Mackay E, Pilling S, Johnson S. Compulsory community treatment to reduce readmission to hospital and increase engagement with community care in people with mental illness: a systematic review and meta-analysis. *Lancet Psychiatry* 2018; published online Oct 31. http://dx.doi.org/10.1016/S2215-0366(18)30382-1.

Literature Search: Community Treatment Orders

Database(s) Ovid XSearch: **PsycINFO** 1806 to December Week 4 2017**, Embase** 1974 to 2018 Week 01**, Ovid MEDLINE(R) Epub Ahead of Print, In-Process & Other Non-Indexed Citations, Ovid MEDLINE(R) Daily and Ovid MEDLINE(R)** 1946 to 4 January 2018
Search Strategy:

#	Searches	Results
1	community treatment order?.ti,ab,kf,kw,id.	853
2	((CTO or CTOs) adj5 (psychiatr* or mental or SMI or psycho* or schizo* or manic or mania or bipolar or antipsycho* or anti-psycho*)).ti,ab,kf,kw,id.	128
3	(((CTO or CTOs) and community) not chronic total occlusion?).ti,ab,kf,kw,id.	481
4	((involuntary or in-voluntary or compulsory or mandatory or mandate?) adj (outpatient? or out-patient? or community treatment)).ti,ab,kf,kw,id.	783
5	((outpatient? or out-patient?) adj commit*).ti,ab,kf,kw,id.	680
6	(((involuntary or in-voluntary or compulsory or mandatory or mandate?) adj3 commit*) and (community or outpatient? or out-patient?)).ti,ab,kf,kw,id,sh.	562
7	((assisted or supervised) adj (outpatient? or out-patient? or community) adj treatment?).ti,ab,kf,kw,id.	220
8	((conditional adj (release? or discharge?)) and hospital* and (psychiatr* or mental or SMI or psycho* or schizo* or manic or mania or bipolar or antipsycho* or anti-psycho*)).ti,ab,kf,kw,id,jw,hw.	245
9	((court adj2 order*) and community and (psychiatr* or mental or SMI or psycho* or schizo* or manic or mania or bipolar or antipsycho* or anti-psycho*)).ti,ab,kf,kw,id,sh.	223
10	(civil commitment and (outpatient? or out-patient? or OPC)).ti,ab,kf,kw,id.	204
11	OUTPATIENT COMMITMENT/ use psyh	195
12	(COMMUNITY MENTAL HEALTH and (COMMITMENT or COURT REFERRAL?)).hw.	739
13	INVOLUNTARY COMMITMENT/ and (COMMUNITY CARE/ or OUTPATIENT CARE/)	327
14	"COMMITMENT (PSYCHIATRIC)"/	1589
15	(OUTPATIENT? or COMMUNITY).hw.	651427
16	14 and 15	84
17	or/1-13,16	3192

18	remove duplicates from 17	1832

ProQuest Dissertations & Theses Global
Search: "*community treatment order**" *n=98*

Comparison with search terms used by other authors

Search terms highlighted in bold (below) were replicated in the new search, however the acronym *CTO* was qualified (please see lines 2 and 3 (above)) and *IOT*, *AOT* were not used. The phrase '*involuntary commitment*' was also qualified to *community* or *outpatients*. Other synonyms/variant spellings were appended to the new search (where appropriate), (terms not used by the other authors). The new search finds all of the studies included in the other reviews, bar a few references cited by Churchill 2007 (details below).

<u>KISLEY 2017</u>
Kisely S, Campbell LA, O'Reilly R. Compulsory community and involuntary outpatient treatment for people with severe mental disorders. The Cochrane database of systematic reviews. 2017(3):CD004408. DOI: 10.1002/14651858.CD004408.pub5

Search: Cochrane Schizophrenia Group's Specialised Register:
Studies, Intervention field = (**involuntary* or *treatment order* or ****outpatient committment******)

<u>RUGKAS 2016 and MAUGHAN 2014</u>
Rugkasa J. Effectiveness of Community Treatment Orders: The International Evidence. Canadian journal of psychiatry Revue canadienne de psychiatrie. 2016;61(1):15-24.

Maughan D, Molodynski A, Rugkasa J, Burns T. A systematic review of the effect of community treatment orders on service use. Social psychiatry and psychiatric epidemiology. 2014;49(4):651-63

Search terms:
community treatment orders
CTO
mandatory outpatient
involuntary outpatient
outpatient commitment
involuntary commitment
IOT
assisted outpatient treatment

CHURCHILL 2007
Churchill R, Owen G, Swaran S, Hotopf M. International experiences of using community treatment orders. London: Institute of Psychiatry, 2007.

Database: EMBASE, Ovid MEDLINE(R), PsycINFO
Search Strategy:
----
1 **(community adj treatment adj order$)**.mp. [mp=ti, ab, sh, hw, tn, ot, dm, mf, nm, tc, id]
2 *CTO$*.mp. [mp=ti, ab, sh, hw, tn, ot, dm, mf, nm, tc, id]
3 **(mandat$ adj outpatient)**.mp. [mp=ti, ab, sh, hw, tn, ot, dm, mf, nm, tc, id]
4 **(outpatient$ adj commitment)**.mp. [mp=ti, ab, sh, hw, tn, ot, dm, mf, nm, tc, id]
5 **(involuntar$ adj outpatient$)**.mp. [mp=ti, ab, sh, hw, tn, ot, dm, mf, nm, tc, id]
6 (involuntar$ adj commitment$).mp. [mp=ti, ab, sh, hw, tn, ot, dm, mf, nm, tc, id]
7 *IOT*.mp. [mp=ti, ab, sh, hw, tn, ot, dm, mf, nm, tc, id]
8 **(Assisted adj outpatient adj treatment)**.mp. [mp=ti, ab, sh, hw, tn, ot, dm, mf, nm, tc, id]
9 aot.mp. [mp=ti, ab, sh, hw, tn, ot, dm, mf, nm, tc, id]
10 **((civil adj commitment) and OPC)**.mp. [mp=ti, ab, sh, hw, tn, ot, dm, mf, nm, tc, id]
11 or/1-10

References cited by Churchill, not retrieved by the new search (reports/journals not indexed on MEDLINE, Embase or PsycINFO):

1. New York State Office of Mental Health. Kendra's Law. Final report on the status of assisted outpatient treatment. 2005. New York State Office of Mental Health. New York, NY.

2. Scheid-Cook TL. Controllers and controlled: an analysis of participant constructions of outpatient commitment. Sociology of Health & Illness 1993; 15(2):179-198.

3. The Centre for Addiction and Mental Health and the Canadian Mental Health Association. Report on the survey of hospitals' use of community treatment orders and case management services. 2005. Toronto, Ontario, Canada. 2005.

4. Power P. A controlled Study of the clinical effectiveness of community treatment orders in Australia: a 'Mirror-image' analysis. Unpublished. 1992.

5. Rohland BM, Rohrer JE, Richards CC. The long-term effect of outpatient commitment on service use. Administration & Policy in Mental Health 2000; 27(6):383-94.

6. Swartz M, Hiday V, Swanson JW, Wagner HR, Borum R, Burns BJ. Measuring coercion under involuntary outpatient commitment: initial findings from a RCT. Research in the Community and Mental Health 1999; 10:57-77.

Meta-analysis: Readmission Rate Ratio

	Comparison	Time point	Statistics for each study				
			Rate ratio	Lower limit	Upper limit	Z-Value	p-Value
Christy 2009	After CTO v Before CTO	12 Months	0.593	0.565	0.623	-20.906	0.000
Kijellin 2014	After CTO v Before CTO	24 Months	0.955	0.943	0.966	-7.492	0.000
			0.753	0.472	1.201	-1.192	0.233

Rate ratio and 95% CI

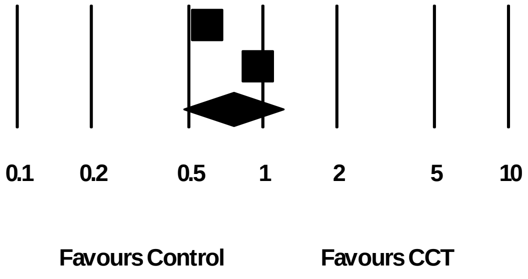

Pre-Post CCT

Meta-analysis: Readmission Risk Ratio

Study name	Comparison	Time point	Statistics for each study				
			Risk ratio	Lower limit	Upper limit	Z-Value	p-Value
Kisely 2004	CTO v No CTO	12 Months	1.250	1.017	1.536	2.123	0.034
Kisely 2005	CTO v No CTO	12 Months	3.230	2.161	4.829	5.716	0.000
			1.977	0.780	5.009	1.437	0.151

Risk ratio and 95% CI

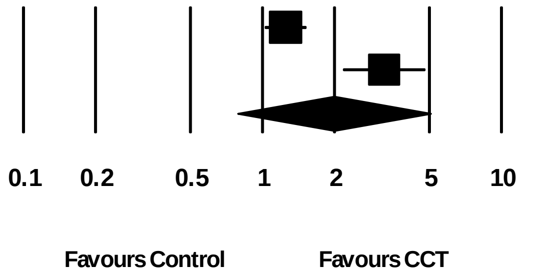

CCT vs Control

Meta-analysis: Inpatient bed days

Study name	Comparison	Time point	Std diff in means	Standard error	Variance	Lower limit	Upper limit	Z-Value	p-Value
Vaughan 2000	After CTO v Before CTO	12 months	-0.139	0.128	0.016	-0.389	0.111	-1.088	0.277
Fernandez 1990	After CTO v Before CTO	36 months	0.151	0.022	0.000	0.108	0.194	6.870	0.000
Zanni 2007	After CTO v Before CTO	24 months	0.258	0.132	0.017	-0.001	0.516	1.954	0.051
Castells-Aulet 2013	During CTO v Before CTO	12 months	0.293	0.149	0.022	0.000	0.585	1.963	0.050
Taylor 2016	After CTO v Before CTO	12 months	0.376	0.036	0.001	0.305	0.447	10.408	0.000
O'Keefe 1997	After CTO v Before CTO	12 months	0.502	0.282	0.079	-0.050	1.054	1.781	0.075
Segal 2006a	After CTO v Before CTO	6 months	0.565	0.059	0.004	0.449	0.681	9.524	0.000
Ozgul 1997	After CTO v Before CTO	24 months	0.595	0.413	0.171	-0.216	1.405	1.439	0.150
Muirhead 2006	During CTO v Before CTO	12 months	0.738	0.151	0.023	0.443	1.034	4.896	0.000
Munetz 1996	During CTO v Before CTO	12 months	0.884	0.331	0.110	0.235	1.534	2.669	0.008
Rohland 2000	After CTO v Before CTO	Variable	1.254	0.172	0.030	0.917	1.591	7.295	0.000
Awara 2013	After CTO v Before CTO	12 months	1.518	0.394	0.156	0.745	2.291	3.848	0.000
O'Brien 2005	After CTO v Before CTO	12 months	1.624	0.326	0.106	0.985	2.264	4.980	0.000
Lera-Catalud 2014	After CTO v Before CTO	12 months	1.697	0.166	0.027	1.372	2.022	10.238	0.000
			0.655	0.101	0.010	0.457	0.853	6.484	0.000

Statistics for each study

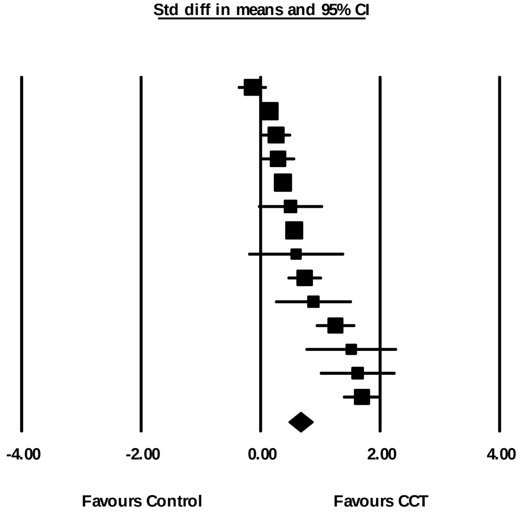

Pre-Post CCT

Meta-analysis: Inpatient bed days

Study name	Comparison	Time point	Std diff in means	Standard error	Variance	Lower limit	Upper limit	Z-Value	p-Value
Zanni 2007	CTO v No CTO	24 months	-0.870	0.157	0.025	-1.178	-0.562	-5.529	0.000
Segal 2006a	CTO v No CTO	6 months	-0.251	0.058	0.003	-0.365	-0.136	-4.297	0.000
Segal 2006b	CTO v No CTO	Variable	-0.205	0.013	0.000	-0.231	-0.179	-15.436	0.000
Kallapiran 2010	CTO v No CTO	12 months	-0.006	0.277	0.077	-0.550	0.537	-0.023	0.982
Burns 2013	CTO v Section 17	12 months	0.084	0.110	0.012	-0.131	0.299	0.768	0.442
Castells-Aulet 2015	CTO v No CTO	24 months	0.170	0.164	0.027	-0.151	0.490	1.038	0.299
Kisely 2013	CTO v No CTO	12 months	0.248	0.026	0.001	0.197	0.300	9.516	0.000
Swartz 1999	CTO v No CTO	12 months	0.456	0.171	0.029	0.121	0.791	2.665	0.008
Hunt 2007	CTO v No CTO	12 months	0.525	0.125	0.016	0.279	0.771	4.186	0.000
Segal 2009	CTO v No CTO	12 months	0.631	0.131	0.017	0.375	0.888	4.827	0.000
Geller 1998	CTO v No CTO	NR	1.061	0.347	0.120	0.382	1.740	3.062	0.002
			0.128	0.107	0.011	-0.082	0.337	1.191	0.233

Std diff in means and 95% CI

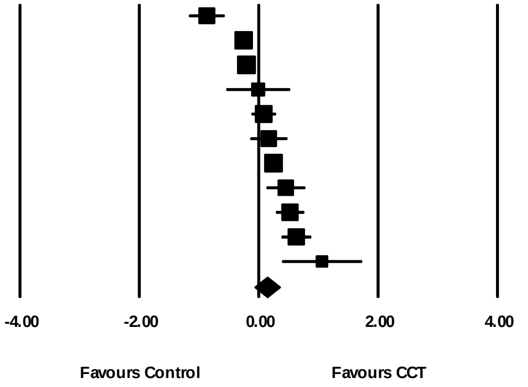

CCT vs Control

Meta-analysis: Use of community services

Study name	Comparison	Time point	Std diff in means	Standard error	Variance	Lower limit	Upper limit	Z-Value	p-Value
Segal 2006a	After CTO v Before CTO	6 months	0.263	0.058	0.003	0.149	0.378	4.506	0.000
Muirhead 2006	During CTO v Before CTO	12 months	0.663	0.150	0.022	0.369	0.956	4.422	0.000
Rohland 2000	During CTO v Before CTO	12 months	0.757	0.163	0.026	0.439	1.076	4.656	0.000
O'Brien 2009	After CTO v Before CTO	12 months	0.932	0.162	0.026	0.614	1.250	5.736	0.000
Munetz 1996	During CTO v Before CTO	12 months	1.258	0.346	0.120	0.580	1.936	3.635	0.000
O'Brien 2005	During CTO v Before CTO	12 months	2.550	0.691	0.477	1.196	3.905	3.691	0.000
			0.832	0.192	0.037	0.455	1.209	4.322	0.000

Std diff in means and 95% CI

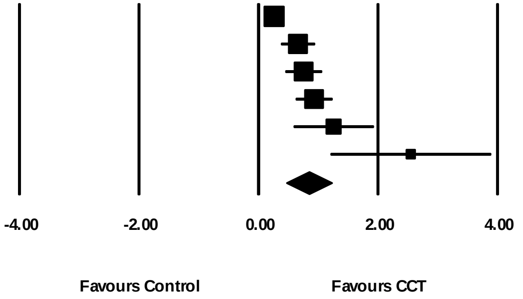

Pre-Post CCT

Meta-analysis: Use of community services

	Comparison	Time point	Std diff in means	Standard error	Variance	Lower limit	Upper limit	Z-Value	p-Value
Hunt 2007	CTO v No CTO	12 months	-0.402	0.115	0.013	-0.628	-0.176	-3.488	0.000
Wagner 2003	CTO v No CTO	12 months	0.056	0.123	0.015	-0.185	0.297	0.454	0.650
Burns 2013	CTO v No CTO	12 months	0.191	0.110	0.012	-0.024	0.406	1.737	0.082
Kisely 2013	CTO v No CTO	12 months	0.281	0.026	0.001	0.230	0.332	10.743	0.000
Pollack 2005	CTO v No CTO	36 months	0.314	0.138	0.019	0.043	0.584	2.272	0.023
Segal 2006a	CTO v No CTO	6 months	0.479	0.059	0.003	0.364	0.595	8.122	0.000
Segal 2006b	CTO v No CTO	Variable	0.629	0.014	0.000	0.603	0.656	46.550	0.000
Hidday 1989	CTO v No CTO	6 months	0.744	0.170	0.029	0.410	1.078	4.365	0.000
Hidday 1987	CTO v No CTO	6 months	1.322	0.174	0.030	0.981	1.663	7.604	0.000
			0.384	0.099	0.010	0.190	0.578	3.873	0.000

Std diff in means and 95% CI

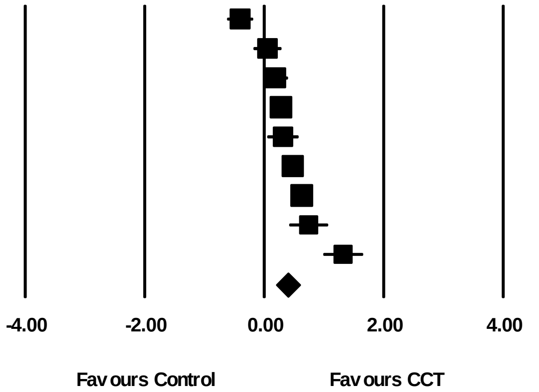

CCT vs Control

Meta-analysis: Treatment adherence

Study name	Comparison	Time point	Std diff in means	Standard error	Variance	Lower limit	Upper limit	Z-Value	p-Value
Dye 2012	After CTO v Before CTO	24 months	1.889	0.380	0.145	1.144	2.635	4.968	0.000
Rawala 2014	After CTO v Before CTO	6 months	2.016	0.383	0.147	1.266	2.767	5.266	0.000
O'Keefe 1997	After CTO v Before CTO	12 months	2.427	0.365	0.134	1.711	3.144	6.641	0.000
			2.120	0.217	0.047	1.694	2.545	9.766	0.000

Statistics for each study

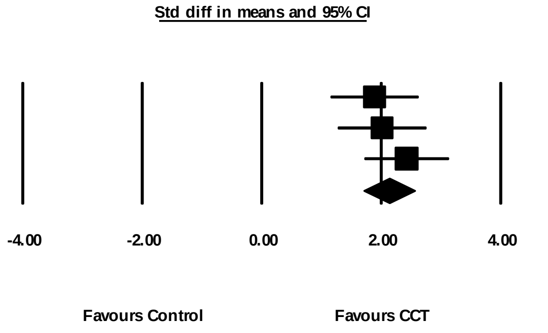

Pre-Post CCT

Meta-analysis: Treatment adherence

Study name	Comparison	Time point	Std diff in means	Standard error	Variance	Lower limit	Upper limit	Z-Value	p-Value
Van Doorn 2010	CTO v No CTO	7 or more months	0.182	0.021	0.000	0.140	0.223	8.559	0.000
Hidday 1987	CTO v No CTO	6 months	1.827	0.571	0.326	0.707	2.946	3.199	0.001
			0.905	0.817	0.667	-0.695	2.506	1.108	0.268

Statistics for each study

Std diff in means and 95% CI

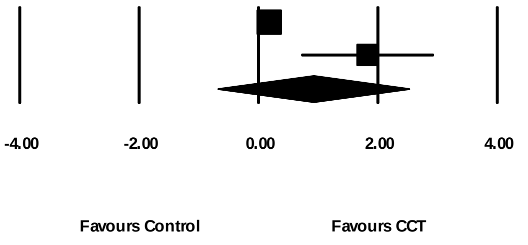

CCT vs Control

Appendix 3

CCT-No CCT Comparison

Outcome	Studies Included	K	Effect Size [a]	95% CI	p-value	Heterogeneity (I^2)
Readmission to Hospital	Both	17	-0·14	-0·41,0·14	·3358	98·06
	RCT	3	-0·08	-0·26,0·41	·6542	69·86
	Non-randomised	14	-0·18	-0·49,0·12	·2418	98·33
Inpatient bed days	Both	11	0·13 [a]	-0·08, 0·34	·2335	97·18
	RCT	2	0·25 [a]	-0·11, 0·61	·1805	70·11
	Non-randomised	9	0·10 [a]	-0·14, 0·33	·4112	97·65
Use of community services	Both	9	0·38 [a]	0·19, 0·58	·0001	96·92
	RCT	2	0·13	-0·03,0·29	·1099	0
	Non-randomised	7	0·46 [a]	0·24, 0·67	<·0001	97·44

[a]*Standard Difference In Means*

Regression of Std diff in means on year of publication

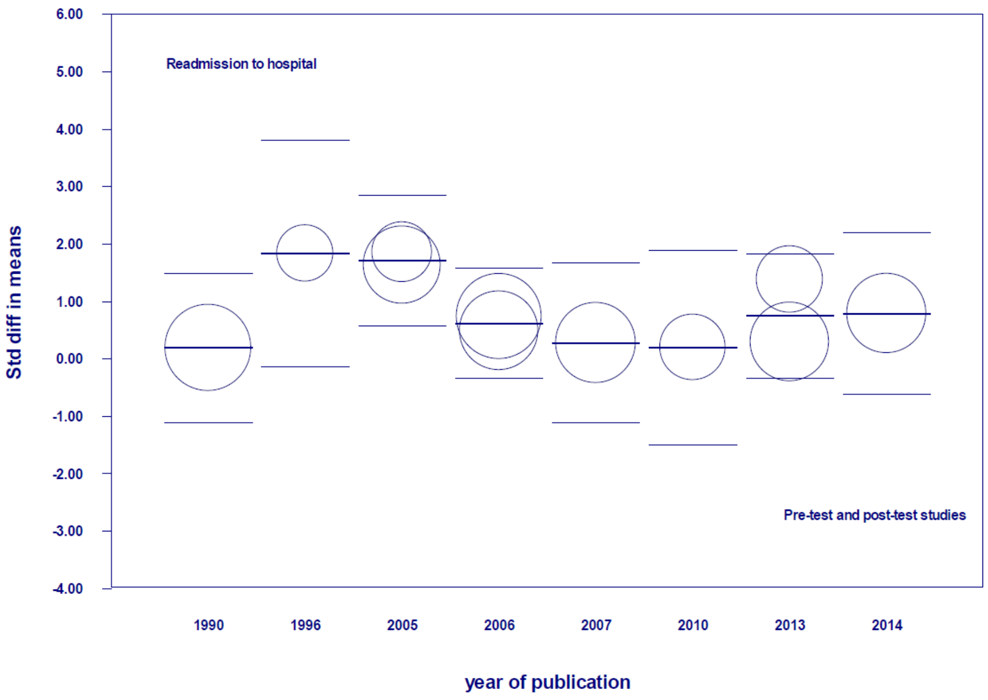

Regression of Std diff in means on year of publication

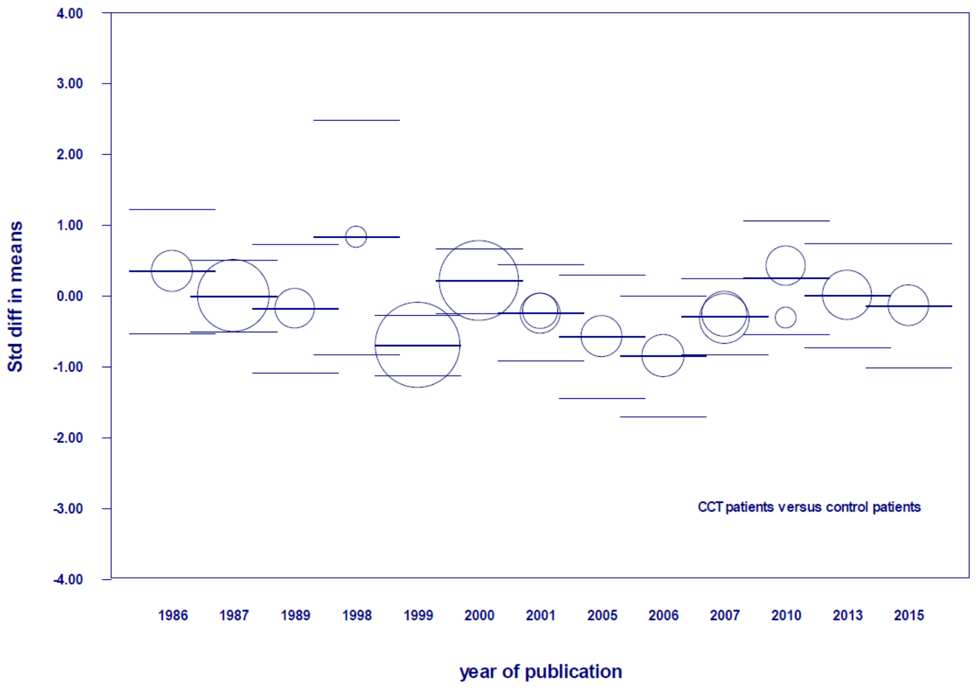

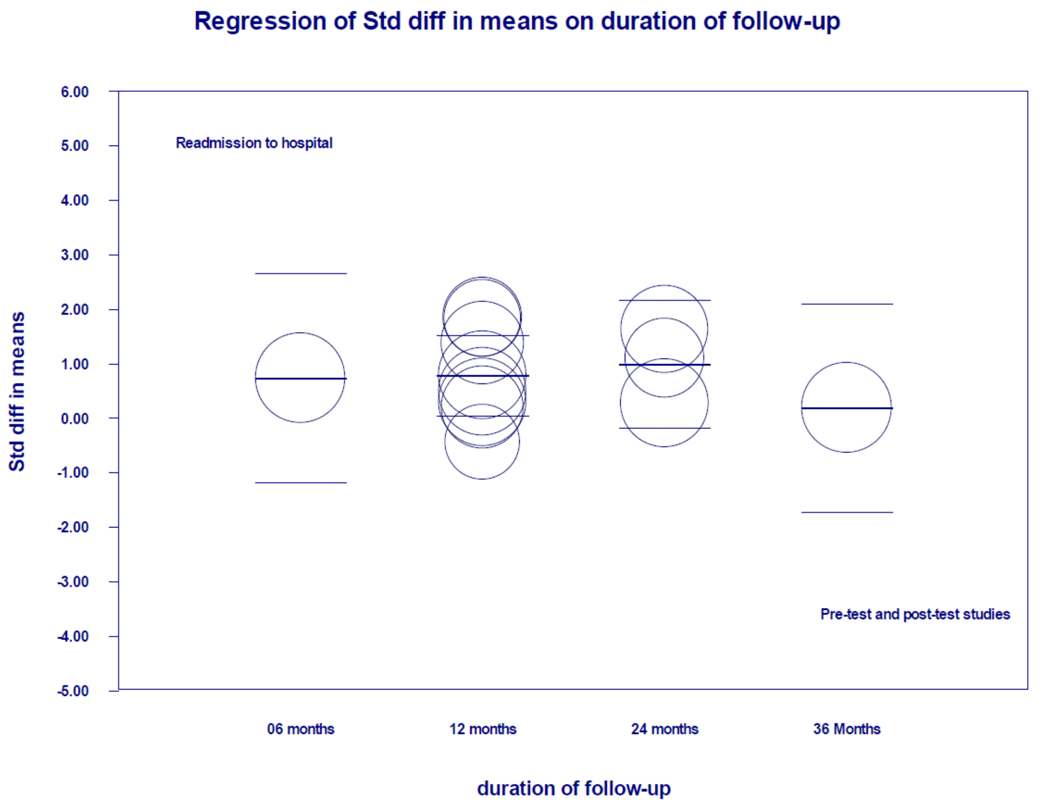
Regression of Std diff in means on duration of follow-up
Readmission to hospital
Pre-test and post-test studies
Std diff in means
6.00
5.00
4.00
3.00
2.00
1.00
0.00
-1.00
-2.00
-3.00
-4.00
-5.00
06 months
12 months
24 months
36 Months
duration of follow-up

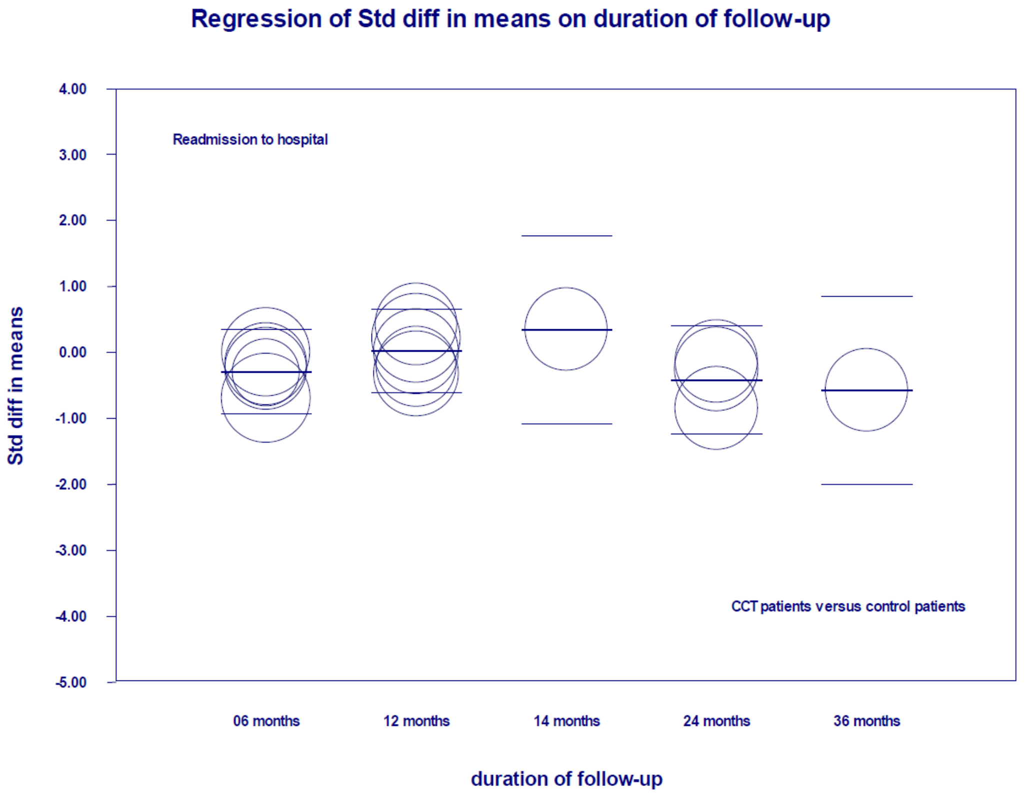
Regression of Std diff in means on duration of follow-up
Readmission to hospital
CCT patients versus control patients
Std diff in means
4.00
3.00
2.00
1.00
0.00
-1.00
-2.00
-3.00
-4.00
-5.00
06 months
12 months
14 months
24 months
36 months
duration of follow-up

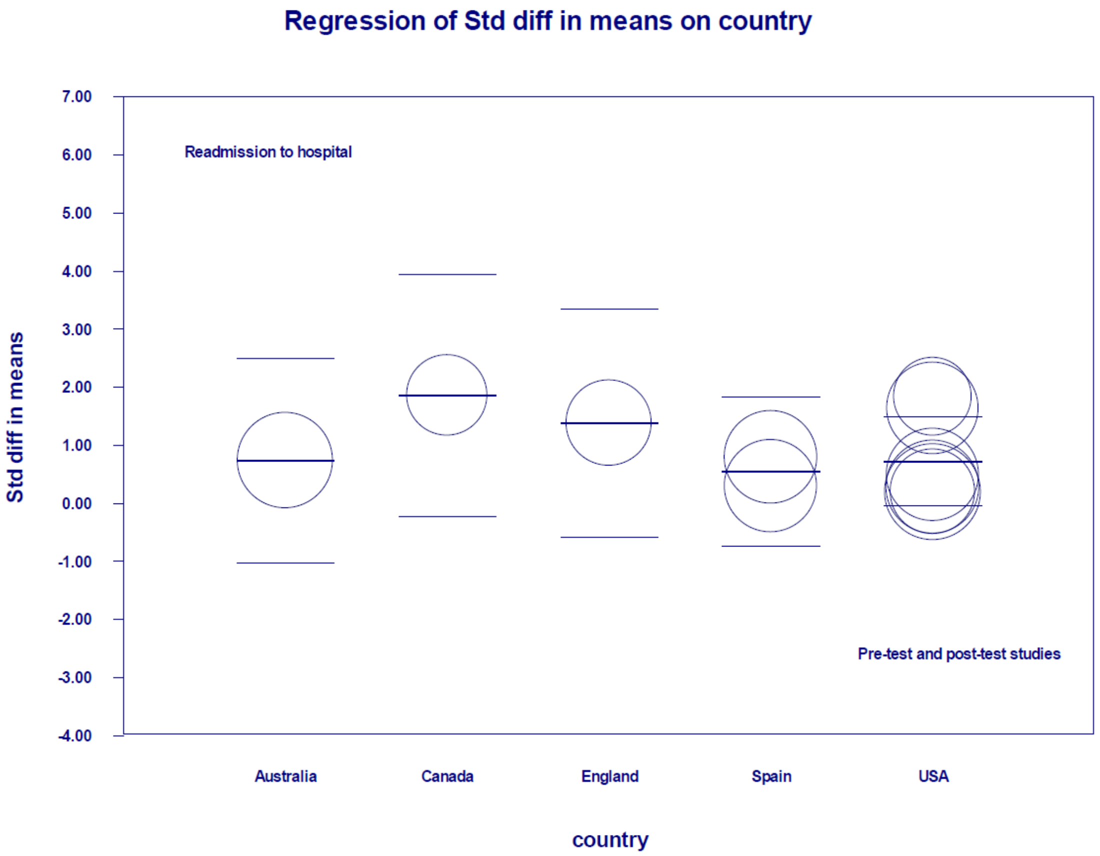
Regression of Std diff in means on country
Readmission to hospital
Pre-test and post-test studies
Std diff in means
7.00
6.00
5.00
4.00
3.00
2.00
1.00
0.00
-1.00
-2.00
-3.00
-4.00
Australia
Canada
England
Spain
USA
country

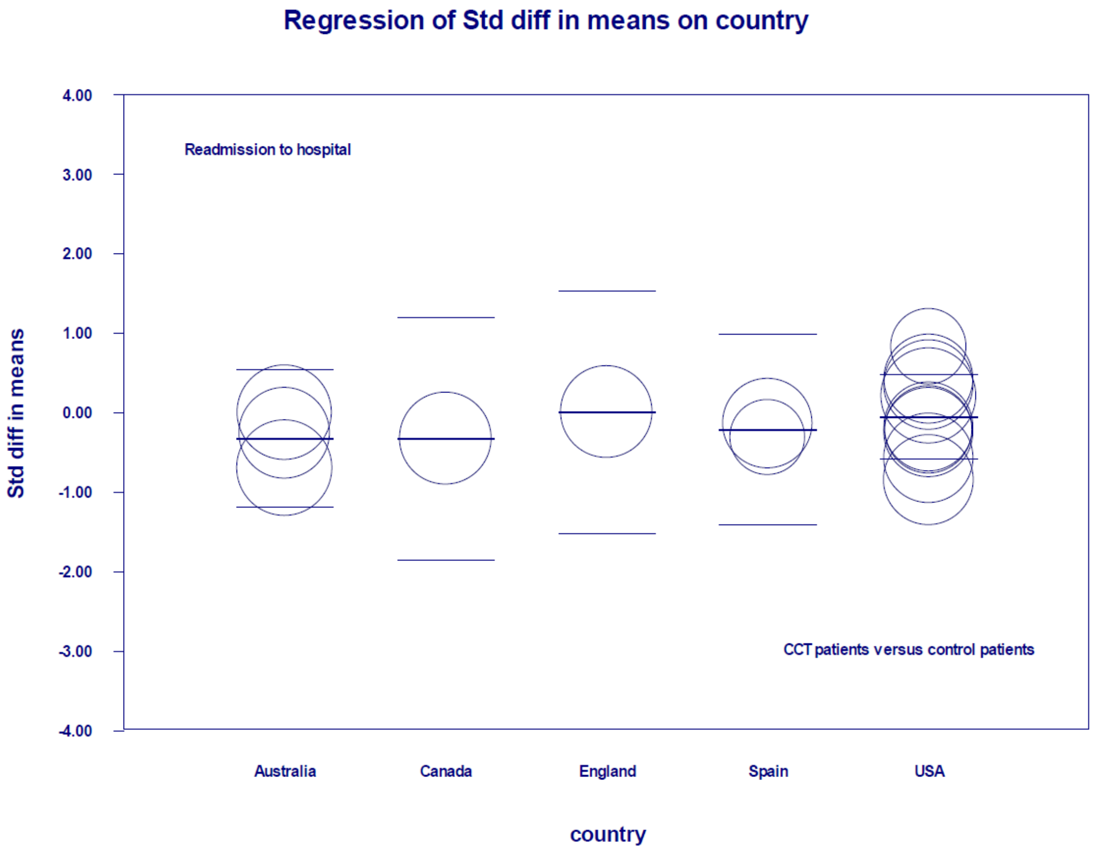
Regression of Std diff in means on country
Readmission to hospital
CCT patients versus control patients
Std diff in means
4.00
3.00
2.00
1.00
0.00
-1.00
-2.00
-3.00
-4.00
Australia
Canada
England
Spain
USA
country

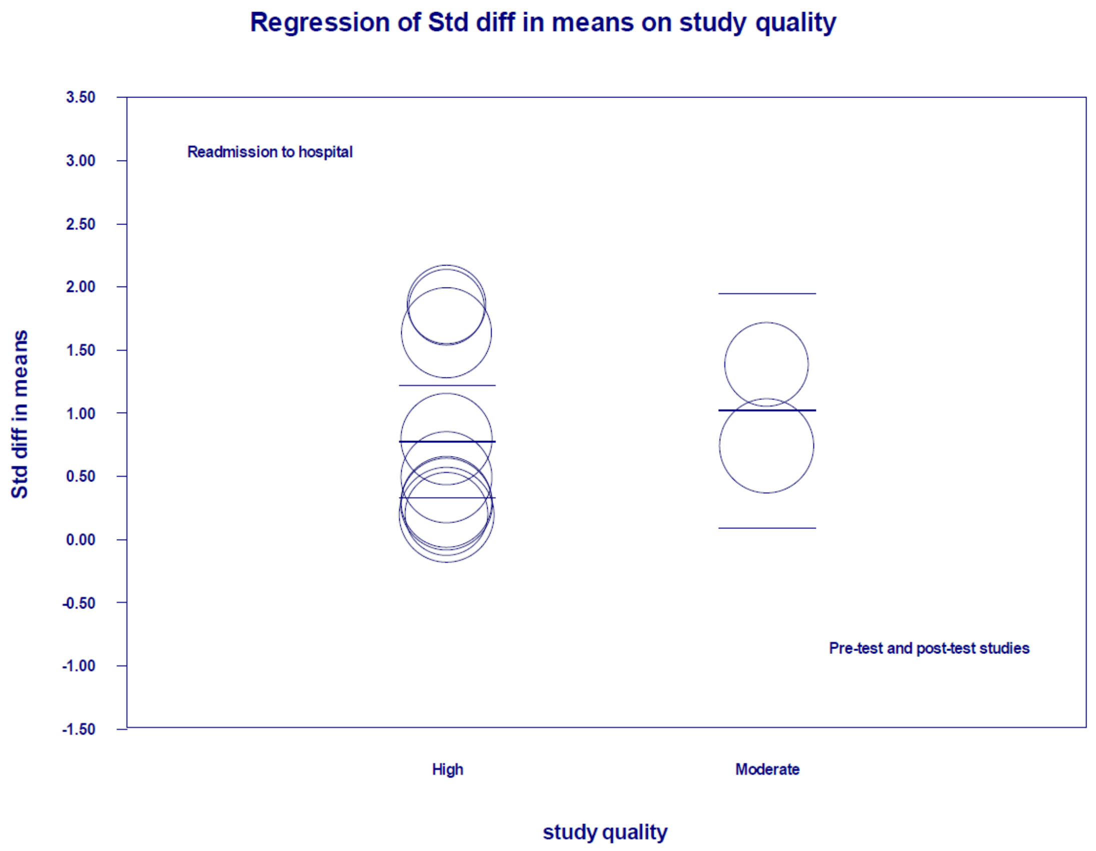
Regression of Std diff in means on study quality
Readmission to hospital
Pre-test and post-test studies
Std diff in means
3.50
3.00
2.50
2.00
1.50
1.00
0.50
0.00
-0.50
-1.00
-1.50
High
Moderate
study quality

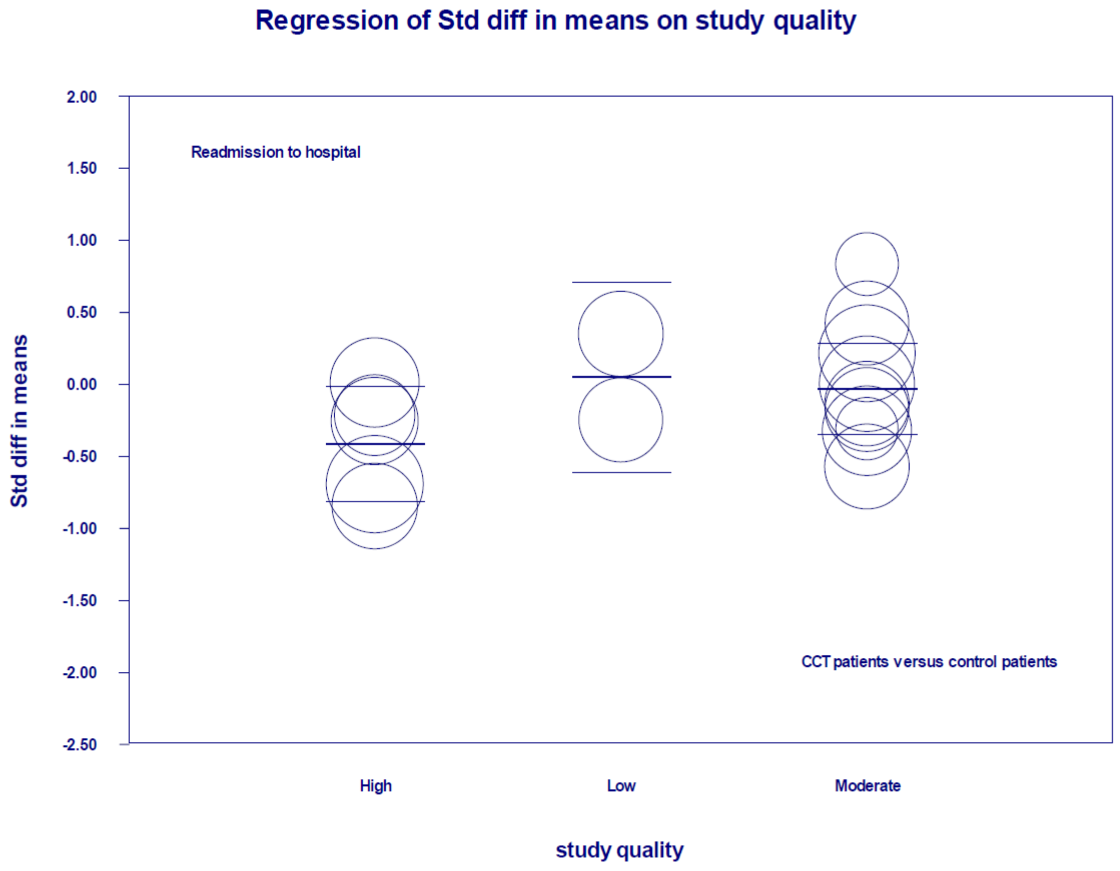
Regression of Std diff in means on study quality
Readmission to hospital
CCT patients versus control patients
Std diff in means
2.00
1.50
1.00
0.50
0.00
-0.50
-1.00
-1.50
-2.00
-2.50
High
Low
Moderate
study quality

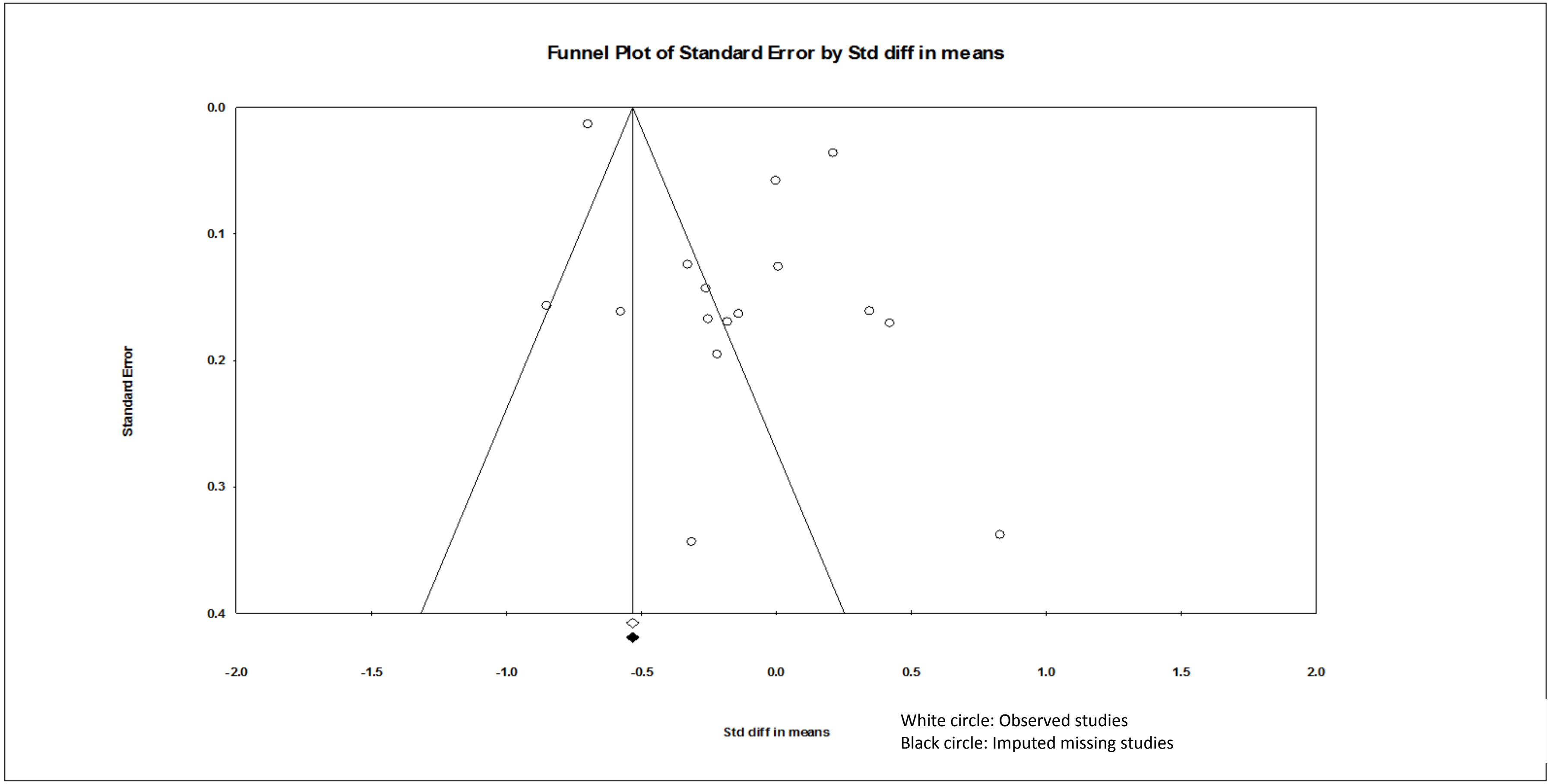

Readmission, CCT vs no CCT

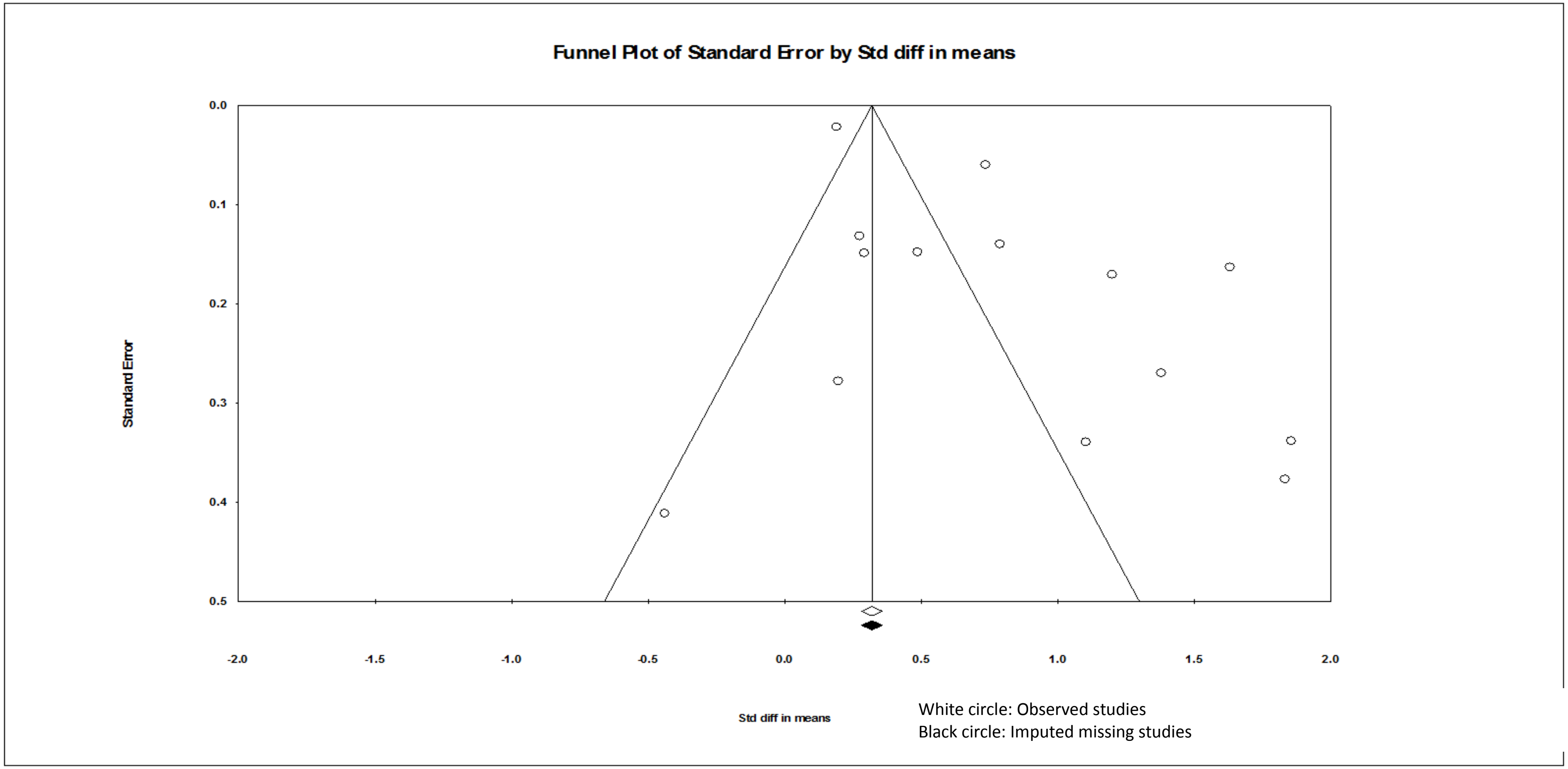

Readmission, Pre-Post designs

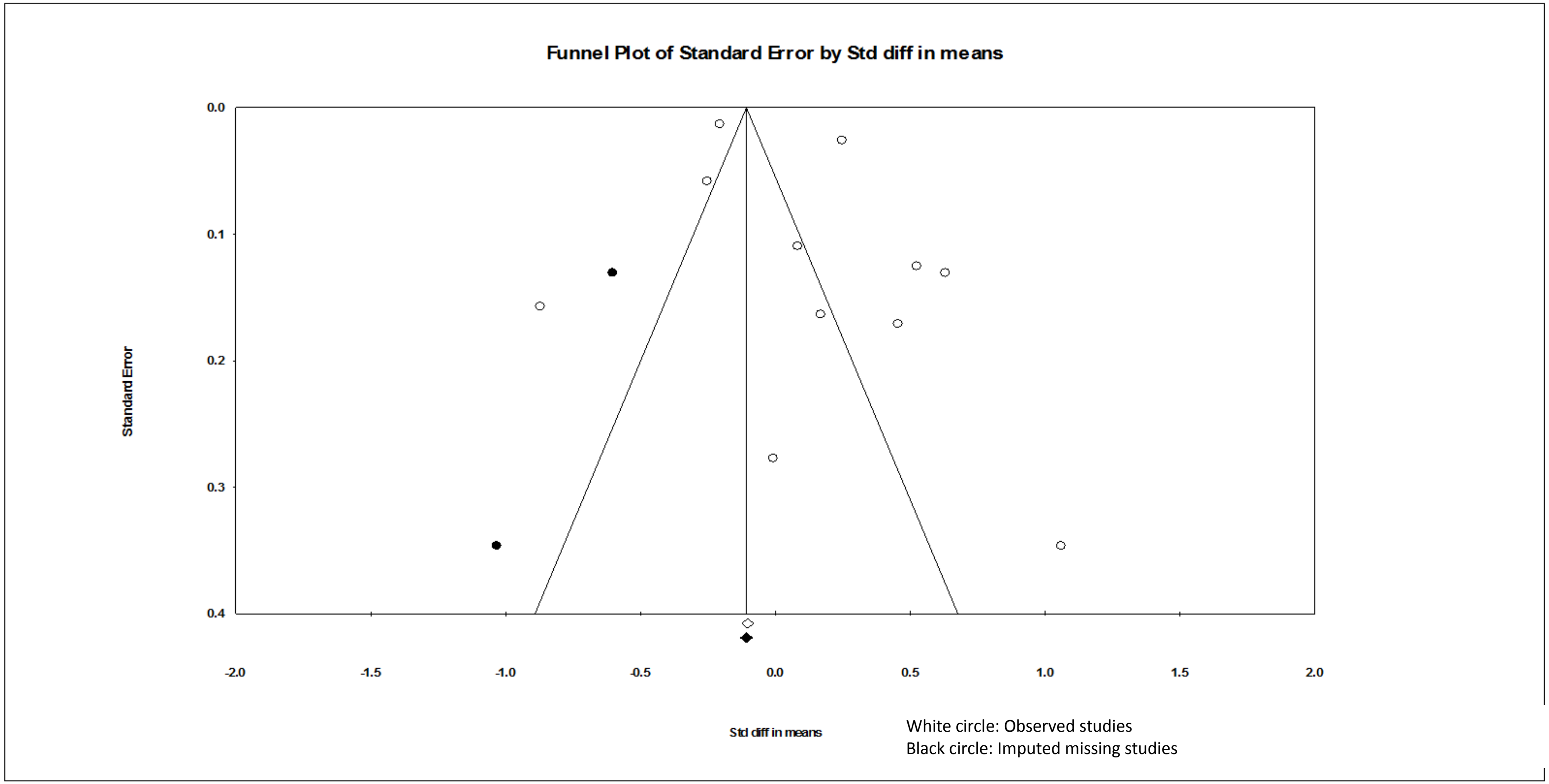

Inpatient bed days, CCT v no CCT

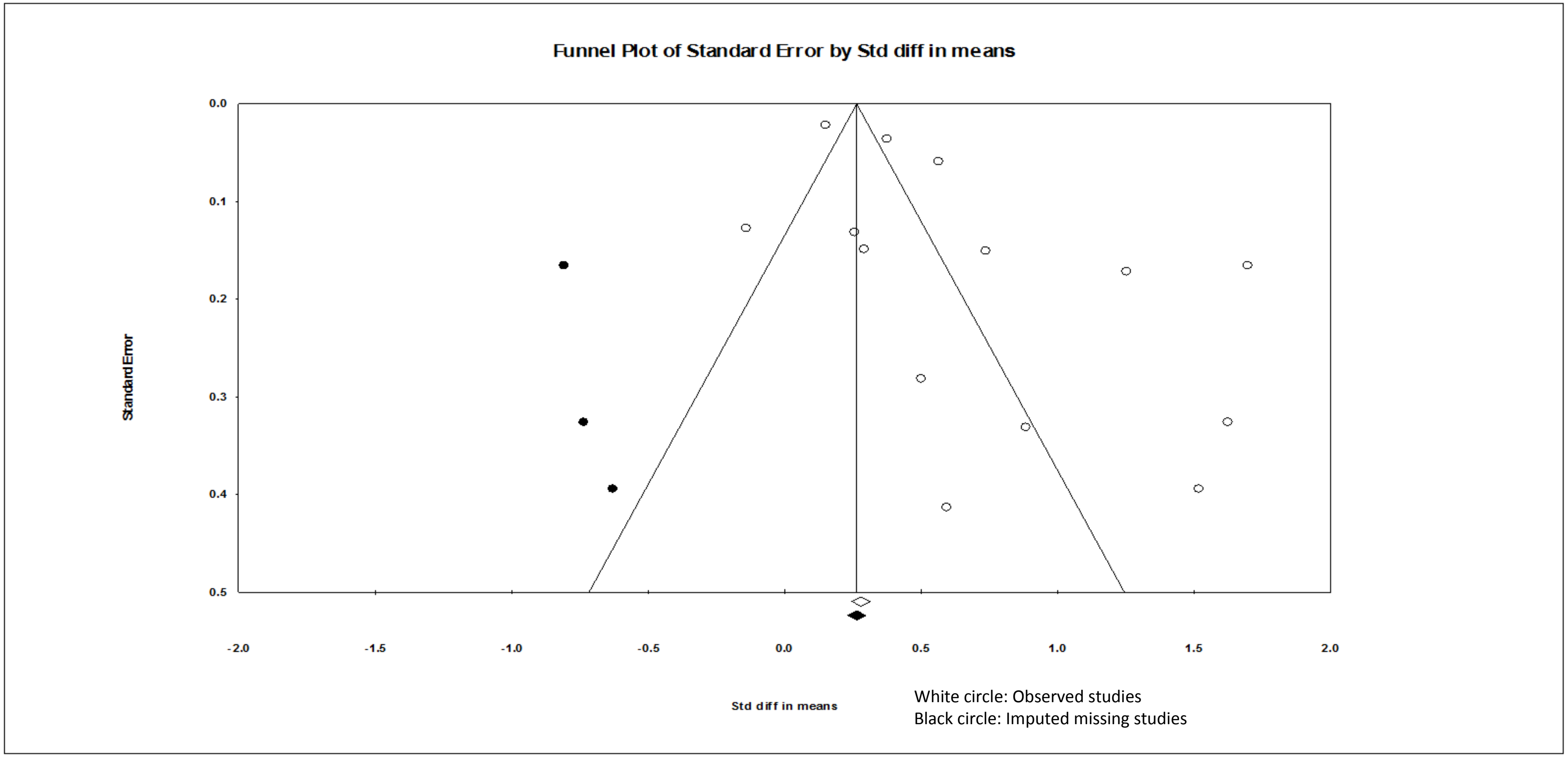

Inpatient bed days, Pre-Post designs

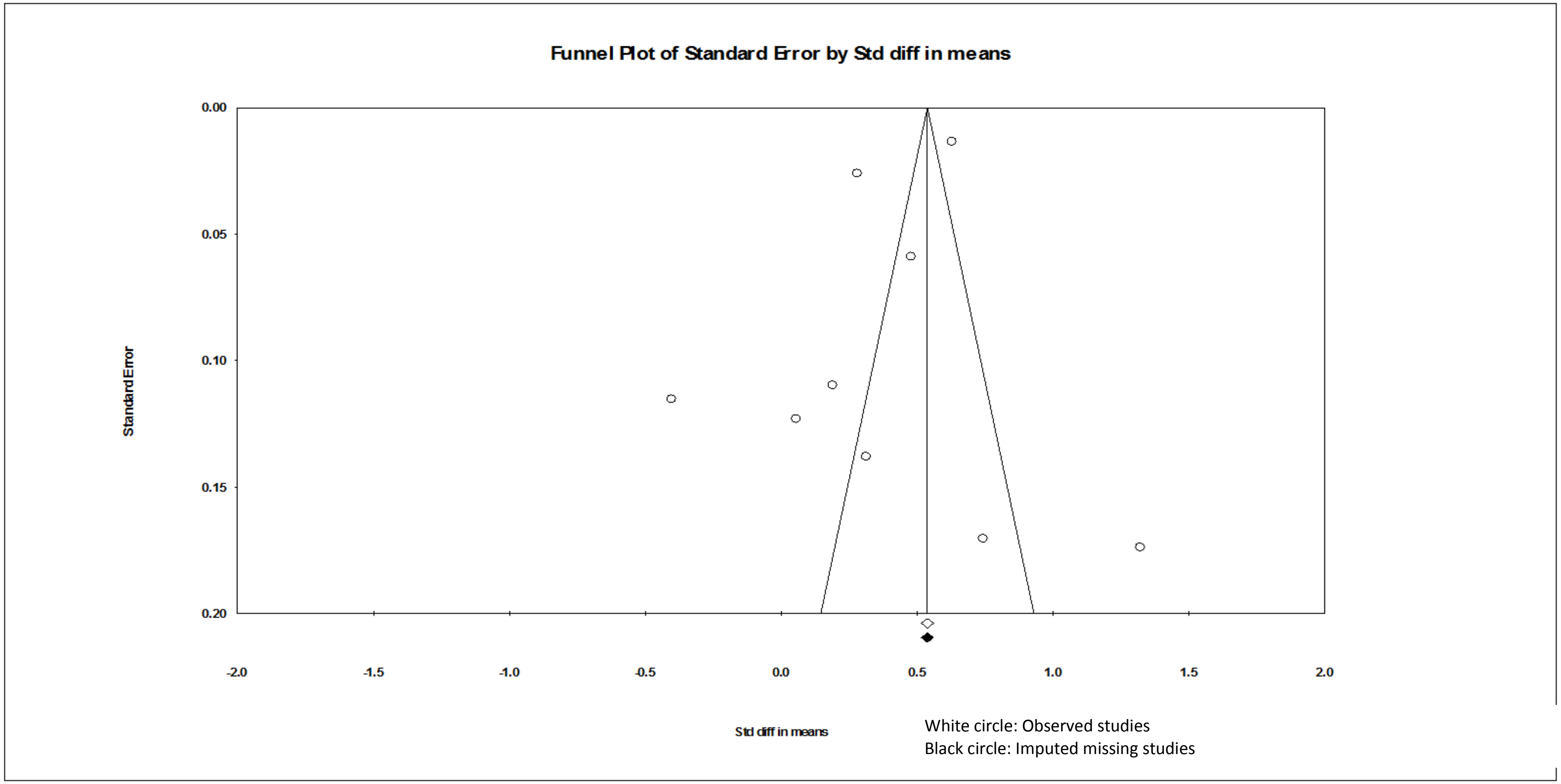

Use of community services, CCT vs no CCT

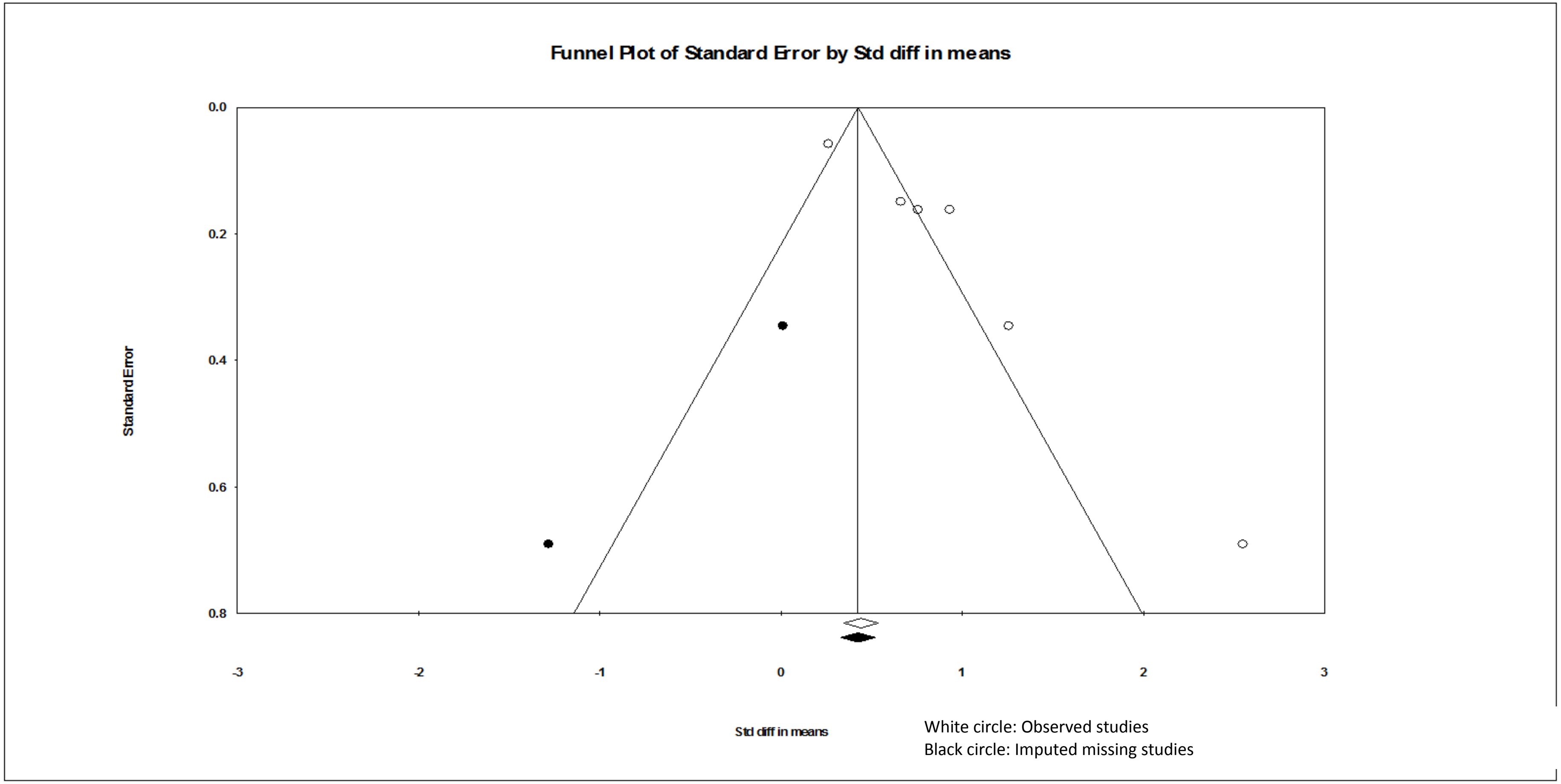

Use of community services, Pre-post designs

Treatment adherence, CCT vs no CCT-insufficient data.

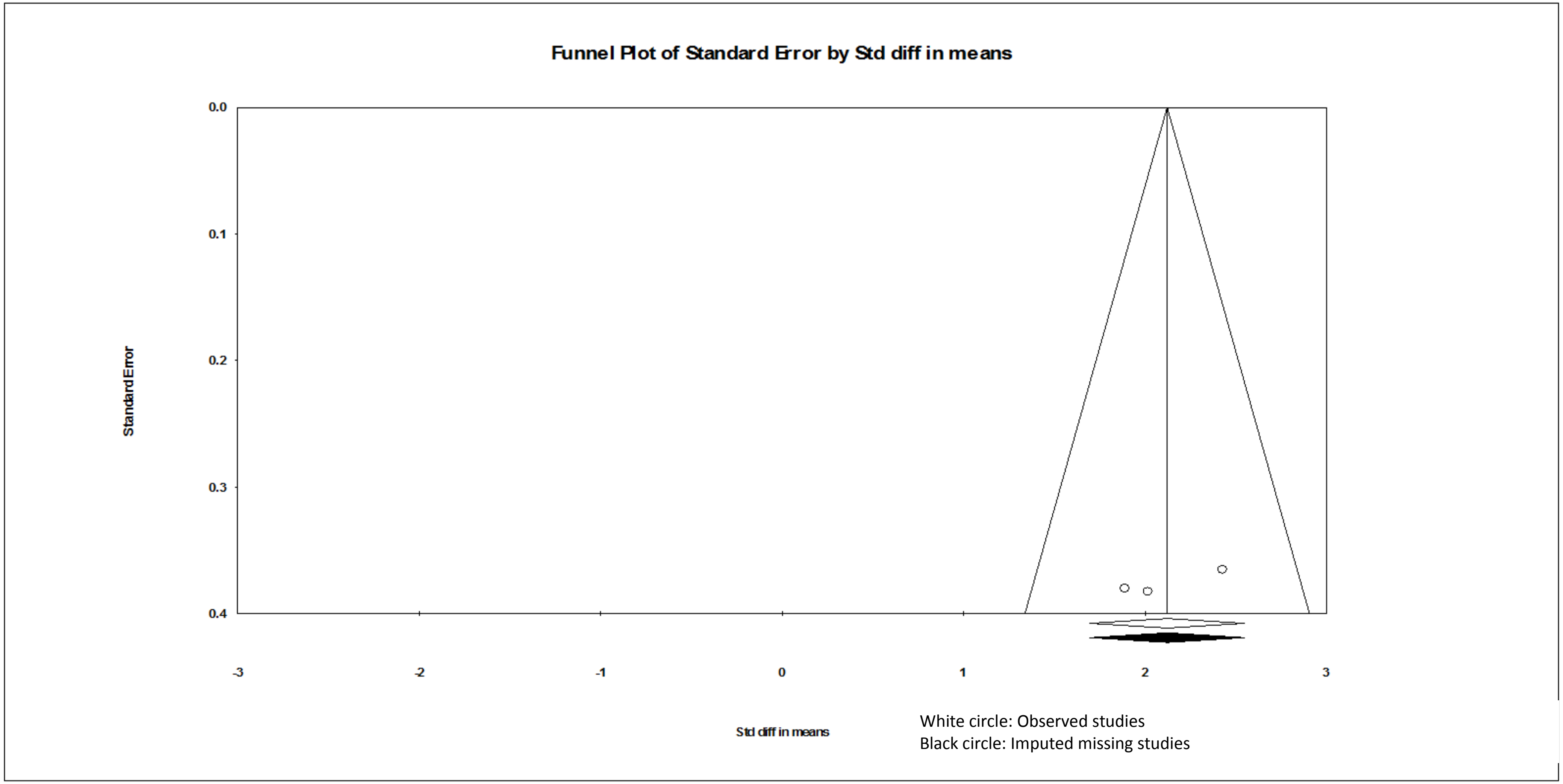

Treatment adherence, Pre-post designs
